# Supplementary material for: Tuberculosis notification in a private tertiary care teaching hospital in South India: a mixed-methods study
Source: BMJ Open. 2019 Feb 5;9(2):e023910. doi: 10.1136/bmjopen-2018-023910 (PMC6377518; doi:10.1136/bmjopen-2018-023910)
Supplement: Author's manuscript [file bmjopen-2018-023910.draft_revisions.pdf]

BMJ Open

BMJ Open is committed to open peer review. As part of this commitment we make the peer review history of every article we publish publicly available.

When an article is published we post the peer reviewers' comments and the authors' responses online. We also post the versions of the paper that were used during peer review. These are the versions that the peer review comments apply to.

The versions of the paper that follow are the versions that were submitted during the peer review process. They are not the versions of record or the final published versions. They should not be cited or distributed as the published version of this manuscript.

BMJ Open is an open access journal and the full, final, typeset and author-corrected version of record of the manuscript is available on our site with no access controls, subscription charges or pay-per-view fees (http://bmjopen.bmj.com).

If you have any questions on BMJ Open's open peer review process please email editorial.bmjopen@bmj.com

BMJ Open

Tuberculosis Notification in a Private Tertiary Care Teaching Hospital in South India: a Mixed Methods Study

Journal:	*BMJ Open*
Manuscript ID	bmjopen-2018-023910
Article Type:	Research
Date Submitted by the Author:	08-May-2018

Complete List of Authors:	Siddaiah, Archana; St John's National Academy of Health Sciences, Community Health<br>Ahmed, Naseer; St John's Research Institute<br>Kumar, Ajay; International Union Against Tuberculosis and Lung Disease (The Union)<br>D'Souza, George; St John's National Academy of Health Sciences<br>Wilkinson, Ewan; University of Chester<br>Mong Mong, Thae ; Department of Medical Research<br>Rodrigues, Rashmi; St John's National Academy of Health Sciences, Community Health; Karolinska Institute, Public Health

Keywords:	operational research, TB notification, Private Medical College, Mixed Methods research, QUALITATIVE RESEARCH

SCHOLARONE™ Manuscripts

For peer review only

Tuberculosis Notification in a Private Tertiary Care Teaching Hospital in South India: a Mixed Methods Study

Authors

Archana Siddaiah[1], Mohammad Naseer Ahmed[2], Ajay M V Kumar[3,4], George D'Souza[5], Ewan Wilkinson[6], Thae Maung Maung[7], Rashmi Rodrigues[1,8,9]

Affiliations

[1]Department of Community Health, St John's Medical College, Bengaluru, India

[2]St John's Research Institute, Bengaluru, India

[3]International Union Against Tuberculosis and Lung Disease, Paris, France

[4]International Union Against Tuberculosis and Lung Disease, New Delhi, India

[5]Department of Pulmonary Medicine, St John's Medical College, Bengaluru, India

[6]Institute of Medicine,University of Chester, CH2 1BR, United Kingdom

[7]Department of Medical Research, Ministry of Health and Sports, Myanmar

[8]Karolinska Institutet, Stockholm, Sweden

[9]Fellow, Wellcome Trust/ DBT India Alliance

Full address

Dr Archana Siddaiah, MBBS, MD
St John's Medical College Hospital
Sarjapur road, John Nagar
Kormangala
Bengaluru 560034
India
Email ID: archanapink@yahoo.com; archana.s@stjohns.in

Dr Mohammad Naseer Ahmed, MBBS
St John's Research Institute
Sarjapur road, John Nagar
Kormangala
Bengaluru
India
Email ID: ahmednaseer1992@gmail.com

Dr Ajay M V Kumar, MBBS MD
International Union Against Tuberculosis and Lung Disease (The Union)
68Boulevard Saint-Michel
F-75006 Paris
France
Email ID: akumar@theunion.org;sathyasaakshi@gmail.com
ORCID id: 0000-0003-1011-8531

For peer review only - http://bmjopen.bmj.com/site/about/guidelines.xhtml

Dr George D'Souza, MD DNB
Department of Pulmonary Medicine
St John's Medical College
Sarjapur road, John Nagar
Kormangala
Bengaluru 560034
India
Email ID: dsouza.ga1975@gmail.com

Professor Ewan Wilkinson, MBCHB FFPH
16 Church Meadow Lane
Wirral
CH60 4SB
United Kingdom
Email ID: ewanwilkinson@gmail.com
ORCID ID: 0000-0002-2167-8756

Dr Thae Mong Mong, MBBS MSc
Department of Medical Research
No.5, Ziwaka Road
Dagon Township
Yangon 11191
Myanmar
Email ID: themgmg.dr@gmail.com

Dr Rashmi Rodrigues, MBBS MD PhD
Department of Community Health
St John's Medical College
Sarjapur road, John Nagar
Kormangala
Bengaluru 560034
India
Email ID: rashmijr@gmail.com

Correspondence to:
Dr Archana Siddaiah, MBBS MD
No 321 Annex 1
St John's Medical College Hospital
Sarjapur road, John Nagar
Kormangala
Bengaluru 560034
India
Email ID: archanapink@yahoo.com; archana.s@stjohns.in
Call: (+91) 9873501269

For peer review only - http://bmjopen.bmj.com/site/about/guidelines.xhtml

Word Count:

Main text: **5486**

Summary: **300**

Number of tables: **4**

Number of figures: 2

Number of boxes: 1

Number of references: 25

Key words: operational research; notification; private medical college; mixed-methods research; qualitative research

For peer review only

ABSTRACT

Objectives: India contributes approximately 25% to the 'missing' cases of tuberculosis (TB) globally. Even though ~50% of patients with TB are diagnosed and treated within India's private sector, few are notified to the public healthcare system despite India's mandatory TB notification policy. We therefore undertook this study to assess the proportion notified and the factors affecting TB notifications at a private hospital in South India. We also explored the barriers and probable solutions to TB notification qualitatively from health provider's perspective.

Study setting: Private, tertiary-care, teaching hospital in Bengaluru, South India.

Methodology: This was a mixed-methods study with quantitative and qualitative components. The quantitative component comprised a retrospective review of hospital records between 1st January 2015 and 31st December 2017 to determine TB notification. The qualitative component comprised key informant interviews and focus group discussions, to find out the factors associated with TB notification.

Results: 3820 patients were diagnosed and treated during this period; 885(23.2%) were notified. Notifications of sputum-smear positive patients were significantly more likely, while notifications of children with TB were less likely. Qualitative analysis yielded themes reflecting the barriers to TB notification and their solutions. Themes related to barriers were (i) basic diagnostic procedures and treatment promote notification, (ii) misconceptions regarding notification and its process is common among healthcare providers (iii) Despite efforts Nikshay does not ensure notification of all patients with TB. Similarly, "establishing systems for notification will go a long way in improving notifications" reflected possible solutions to improve TB notification.

Conclusions: Proportion of TB cases notified at the hospital was low. Combined approach from within (managerial) and outside the institution i.e. Revised National Tuberculosis Control Programme (RNTCP) is necessary. Improving awareness among health care providers about the requirement for notifications, implementing a comprehensive notification policy, and establishing a single notification portal could improve notifications.

Strengths and Limitations:

- A mixed methods design where the qualitative component explains the findings from the quantitative component.
- Retrospective nature of the quantitative component ensured that the study did not influence the notifications.
- It is likely that both the proportions notified and the number of patients diagnosed or treated is marginal overestimated.
- The findings are limited by the quality of the records maintained

For peer review only - http://bmjopen.bmj.com/site/about/guidelines.xhtml

BACKGROUND

In the year 2016, approximately 40% of the estimated 10.4 million TB cases were 'missing', i.e., were undiagnosed or unreported.[1] India contributes approximately 25% to the 'missing' cases globally.[1,2] Finding these 'missing' cases and treating them successfully is vital to ending TB by 2030, as envisaged by the United Nations Sustainable Development Goals.[3,4]

Healthcare delivery in India involves both the public and private sectors. Therefore, it is not surprising that many patients with TB are diagnosed and treated within the private healthcare sector.[5] However, few of these patients receive care from, or are notified i.e., reported, to the Revised National Tuberculosis Control Programme (RNTCP).[4,6] Though, mandatory TB notification introduced in 2012 saw a sharp increase in TB notifications, notifications from the private sector continues to be low.[4,6–10] Disease notification facilitates TB care through the RNTCP by improving the estimates of disease prevalence that are essential to planning, monitoring, and evaluation of healthcare programmes. Lack of time, low awareness regarding notification, concern towards breaching patient confidentiality, operational complexities in notifying along with lack of trust in the public sector contribute to poor TB notifications from the private sector.[11,12]

The information on the extent of notification from private tertiary care teaching facilities is limited. This study was designed to determine the proportion of TB cases notified and the factors that affect notification at such a private tertiary care teaching healthcare facility in Karnataka State, South India. The study also explored qualitatively the gaps in the existing notification systems so as to enable the identification and development of strategies to improve notification.

For peer review only

For peer review only - http://bmjopen.bmj.com/site/about/guidelines.xhtml

METHODS

Study design

A mixed-methods study comprising retrospective review of records to quantitatively assess the proportions of patients with TB notified, and a qualitative component to identify barriers to TB notification was used.

Study setting

The study was conducted at a private tertiary level teaching hospital in Bengaluru, Karnataka state in South India. The hospital has a 1250 bedded inpatient facility and caters to approximately 2000 outpatients, from diverse backgrounds, everyday. A network of laboratory, pathology, and radiology services support the clinical departments at the hospital. TB specific microbiological services available are microscopy, GenXpert MTB/RIF®, solid culture, and liquid TB culture and DST (MGIT).

A computerised information system for these services and the pharmacy exist at the hospital. The Medical Records department (MRD) compiles and maintains inpatient and outpatient hospital records in paper format. Inpatient records are available electronically and outpatient records are available in paper format.

Management of TB at the hospital

The RNTCP through a public-private partnership has established a Designated Microscopy Centre (DMC) for sputum smear microscopy and a Directly Observed Treatment (DOT) Center for providing treatment to TB patients at no cost. The RNTCP staffs the DMC with a laboratory technician (LT), and the DOT center with a medical officer (position currently vacant) and a TB Health Visitor (TBHV).

For peer review only - http://bmjopen.bmj.com/site/about/guidelines.xhtml

For peer review only

When diagnosed with TB at any of the clinical departments at the hospital, patients can choose to avail anti-tubercular treatment (ATT) either through the DOT center, at no cost, or through the hospital's pharmacy, for a cost. The patient's physician guides the patient's choice of treatment on a case by case basis.

Notification

Irrespective of the source of treatment, all patients with TB that are diagnosed or treated at the hospital are expected to be referred to the DOT center for registration with the RNTCP and subsequent notification via the online notification portal Nikshay (an electronic recording and reporting system).[13] In the study hospital notification of TB cases is the responsibility of the TBHV.

Study Population

Quantitative component

All patients diagnosed with TB and/or treated for TB from 1st January 2015 to 31st December 2016 comprised the study population. For this study, a case of TB was defined as (i) a patient with microbiologically confirmed TB using microscopy, bacterial culture, and/or GenXpert MTB/RIF® OR (ii) a patient with histopathological or radiological findings suggestive of TB, irrespective of microbiological confirmation, OR (iii) a patient who availed ATT from the hospital's pharmacy identified through the pharmacy information system (PIS).

Qualitative component

Health care providers caring for TB patients from various departments including clinicians, staff nurse, researchers, RNTCP LT, and RNTCP TBHV were interviewed in-depth.

For peer review only - http://bmjopen.bmj.com/site/about/guidelines.xhtml

Participants were chosen purposively to include those involved at various points within the TB case management cascade which is depicted in **Figure 1**.

Data sources, variables and data collection procedures

Quantitative component

Details of patients with TB were extracted from multiple sources. Data was first extracted from the inpatient electronic medical records database using the International Classification of Disease 10 (ICD 10) coding for TB (codes A15 to A19). Subsequently data from the histopathology component of the laboratory information system (LIS) was extracted. For this, search terms such as "tuberculosis", "TB" and for possible typographical errors and "lower and upper case formats" (eg., TB or tb) were used as these diagnoses did not follow the ICD 10 coding. Data was similarly extracted from the Radiology Information System (RIS). These comprised reports from Computerised Tomography (CT) and Magnetic Resonance Imaging (MRI). Chest radiographs were not reported in the RIS as physicians review them in the light of clinical evidence for diagnosis. A laboratory or radiology report that read "acid-fast bacilli (AFB) positive" or "MTB detected" or "strongly suggestive of TB" were considered as a case of TB. When in doubt, two physicians reviewed the reports and arrived at a consensus on the diagnosis. The pharmacy information system (PIS) provided data regarding ATT purchased at the hospital's pharmacy.

Further, details of positive reports from sputum microscopy and culture registers were manually extracted and entered into Microsoft (MS) Excel as they were not available in the LIS.

A "master database" for TB patients diagnosed and/or treated in 2015 and 2016 was created using the unique hospital number (allocated to a patient at registration in the hospital) to

For peer review only - http://bmjopen.bmj.com/site/about/guidelines.xhtml

match records and delete duplicate records in the various databases (PIS, LIS, RIS and manual registers).

A “notification database” for TB patients notified was also created. For this, data from the RNTCP register at the DOT center of the hospital was entered into MS Excel. This was merged with data extracted from Nikshay portal. Patients diagnosed in late 2016 but got notified in the first quarter of 2017 were also incorporated into this database.

In order to identify the proportions of TB cases notified the “master database” was matched with the “notification database”, using the VLOOKUP function in MS Excel. The patient’s name was used as the primary matching variable. Records with a typographical mismatch in the patient’s name were matched using a perfect match for ‘sex’ within an age range of ±3 years. Flowchart of data sources is depicted in **Figure 2.**

Qualitative Component

We conducted 11 in-depth interviews (IDI) with various healthcare providers and one focus group discussion (FGD) only with the nursing staff. The first author (AS), a physician trained in qualitative research, conducted the interviews. Two of the interviews were conducted in the local language, Kannada, and the rest in English. All interviews were audio recorded. A rapporteur made field notes during the interviews. After each interview, the key points were summarized and verified with the participants for validation. Data saturation guided the sample size. Each IDI lasted for 15-45 minutes and the FGD lasted for an hour.

Data Analysis

Quantitative component

For peer review only - http://bmjopen.bmj.com/site/about/guidelines.xhtml

EpiData (v2.2.2.186, EpiData Association, Odense, Denmark) and Stata (v12.1, Texas, USA) software were used for data analysis. The proportion of TB patients notified was the outcome variable. Associations (unadjusted) between the outcome variable and demographic and clinical characteristics were derived using the Chi square test. All bivariate associations with a ‘p value’<0.20 were included in a log-binomial regression model to obtain adjusted prevalence ratios (PR) with 95% confidence intervals. A ‘p value’<0.05 was considered statistically significant.

Qualitative component

All interviews and field notes were transcribed and translated into Englishfor analysis using the ‘thematic framework approach’. The first and last author (AS, RR) familiarised themselves with a few transcripts and manually coded them. The codes were then compared and categorised based on similarity. This formed the framework for the analysis.[14] The rest of the transcripts were subsequently indexed using the codes generated. Additional codes were added as and when necessary. **(Panel 1)** The data was then summarised and mapped under various subthemes and themes which were reviewed by the rest of the authors for consensus.

Patient and Public Involvement Statement

Patients were not involved in the design or conduct of the study.

Ethics

Ethics approval was obtained from the Institutional Ethics Committee, St. John’s Medical College, Bengaluru, Karnataka State, India and the Ethics Advisory Group of the International Union Against Tuberculosis and Lung Disease, Paris, France. Permission to conduct the study and access hospital records was obtained from the Chief of Medical

For peer review only - http://bmjopen.bmj.com/site/about/guidelines.xhtml

Services at the hospital. Written informed consent was obtained from healthcare providers prior to interviews and included consent to audio-record the interviews.

Panel 1: Thematic framework used for understanding the issues with TB notification at a private tertiary care teaching hospital, Bengaluru, India 2015-2016

1.1 Gaps in the TB notification
1.2 Missing TB patients
1.3 Confidentiality issue
2. Information to doctors
3. Disease disclosure to patients
4.1 TB diagnostic standard operating procedures
4.2 Technical issues associated with TB diagnosis
5.1 Doctors' role in TB notification
5.2 Reporting of TB patients by doctors
5.3 Co-ordination between doctors and DOT center
6.1 Standard operating procedures for TB notification
6.2 Ease of notification
7.1 Policy decisions
8.1Institute's notification policy
8.2 RNTCP notification policy
8.3 Gaps in RNTCP notification policy
9.1Streamlining TB notification
9.2Technological involvement

DOT-Directly Observed Treatment short course; RNTCP-Revised National Tuberculosis Control Programme

RESULTS

Quantitative component

3820 patients were diagnosed with TB and/or treated during the study period of two years. The demographic details of the patients with TB are described in **Table 1**. The median (inter-quartile range) age was 40 (27-56) years and 7% of the patients were children <15 years of age.

About a quarter of the patients received inpatient care and nearly half were inpatients under internal medicine, followed by chest medicine, neurology and paediatrics. About half of the

For peer review only - http://bmjopen.bmj.com/site/about/guidelines.xhtml

patients with TB were identified through the pharmacy database while nearly 25% were identified through the LIS and laboratory registers.

Of the 3820 patients with TB, 885 (23.2%, 95% CI: 21.9-24.5) were notified to the RNTCP. Of those notified 82 (9%) were also recorded in the Nikshay portal. Factors associated with notification are shown in **Table 2.** Notification was significantly lower (unadjusted analysis) in children, inpatients and patients identified through the LIS and PIS. Notification was significantly higher for patients whose diagnosis was confirmed microbiologically (sputum-smear microscopy, culture or GenXpert MTB/RIF®). The final adjusted regression model comprised age and sputum- microscopy as determinants of notification.

Table 1. Demographic profile of patients with TB diagnosed and/or treated from 2015 to 2016 at a private tertiary care teaching hospital in Bengaluru, India

Variable	*Number (%)*	*Notified (%)*
Total	***3820 (100)***	***885 (23·2)***
Age in years		
0-14	264 (6·9)	24 (9·1)
15-24	476 (12·5)	118 (24·8)
25-34	802 (21·0)	166 (20·7)
35-44	670 (17·5)	159 (23·7)
45-54	598 (15·7)	160 (26·8)
55-64	503 (13·2)	129 (25·6)
65 and above	507 (13·3)	129 (25·4)
Sex		
Male	2320 (60·7)	559 (24·1)
Female	1500 (39·3)	326 (21·7)
Residence		
Within state	2362 (61·8)	567 (24·0)
Outside state	1358 (35·5)	293 (21·6)
Not available	100 (2·6)	25(25·0)
Marital status		
Unmarried	1008 (26·4)	183 (18·2)
Married	2604 (68·2)	653 (25·1)
Others	208 (5·4)	49 (23·6)
Year		
2015	2071 (54·2)	482 (23·3)
2016	1749 (45·8)	403 (23·0)
Admission		
Yes	1009 (26·4)	137 (13·6)

For peer review only - http://bmjopen.bmj.com/site/about/guidelines.xhtml

No	2811 (73·6)	748 (26·6)
Department (n=1009)		
Medicine	484 (48·0)	64 (13·2)
Pulmonary Medicine	141 (14·0)	21 (14·9)
Paediatrics	81 (8·0)	16 (19·8)
Neurology	88 (8·7)	5 (5·7)
General Surgery	41 (4·1)	5 (12·2)
Orthopaedics	50 (5·0)	8 (16·0)
Others	124 (12·3)	15 (12·1)
Source of TB patients*		
Sputum microscopy register	747 (19·6)	481 (64·4)
Extrapulmonary TB positive register	124 (3·2)	24 (19·4)
Histopathology database	203 (5·3)	53 (26·1)
Radiology database	92 (2·5)	13 (13·7)
Pharmacy database	1754 (45·9)	341 (19·4)
Culture Register	227 (5·9)	72 (31·7)
GenXpert MTB/RIF® register	91 (2·4)	38 (41·8)
Inpatient database	1009 (26·4)	137 (13·6)

Cumulative percentage may add up to more than 100 since one patient could have tested positive by more than one diagnostic methods

Table 2. Factors associated with TB notification at a private tertiary care teaching hospital in Bengaluru, India from 2015 to 2016

Variable	*Total*	*Notification Number (%)*	*Crude PR (95% CI)*	*P value*	*Adjusted PR (95% CI)*	*P value*
Total	3820	885 (23·2)	-	-	-	-
Age (years)	-	-	-	-	-	-
Children (<15)	264	24 (9·1)	1	-	1	-
Adults (≥ 15)	3556	861 (24·2)	2·6 (1·8-3·9)	0·000*	1·5 (1·0-2·2)	0·039*
Sex	-	-	-	-	-	-
Female	1500	326 (21·7)	1	-	-	-
Male	2320	559 (24·1)	1·1 (0·9-1·2)	-	-	-
Marital status	-	-	-	-	-	-
Unmarried	1008	183 (18·2)	1	-	1	-
Married	2604	653 (25·1)	1·3 (1·1-1·5)	0·000*	1·0 (0·9-1·2)	0·240
Others	208	49 (23·6)	1·2 (0·9-1·7)	0·066	1·1 (0·8-1·4)	0·346
Admission	-	-	-	-	-	-
No	2811	748 (26·6)	1	-	1	-
Yes	1009	137 (13·6)	0·4 (0·4-0·5)	0·000*	1·0 (0·8-1·2)	0·925
Residence	-	-	-	-	-	-
Within state	2362	567 (24·0)	1		-	
Outside state	1358	293 (21·6)	0·8 (0·7-1·0)	0·092	-	-

For peer review only - http://bmjopen.bmj.com/site/about/guidelines.xhtml

Not recorded	100	25 (25·0)	1.0 (0·7-1·4)	0·819	-	-
Year	-	-	-	-	-	-
2015	2071	482 (23·3)	1	-	-	-
2016	1749	403 (23·0)	0·9(0·8-1·1)	0·866	-	-
Sputum smear microscopy	-	-	-	-	-	-
Positive	747	481 (64·4)	4·8 (4·4-5·4)	0·000*	4·7 (4·1-5·3)	0·000*
Others	3073	404 (13·1)	1	-	1	-
EPTB microscopy register	-	-	-	-	-	-
Positive	124	24 (19·4)	0·8 (0·5-1·1)	0·318	-	-
Others	3696	861 (23·3)	1	-	-	-
Culture	-	-	-	-	-	-
Positive	227	72 (31·7)	1·4 (1·1-1·7)	0·001*	1·0 (0·8-1·2)	0·855
Others	3593	813 (22·6)	1		1	
GenXpert MTB/RIF®	-	-	-	-	-	-
Positive	91	38 (41·8)	1·8 (1·4-2·3)	0·000*	1·1 (0·9-1·3)	0·295
Others	3729	847 (22·7)	1	-	1	
Histopathology database	-	-	-	-	-	-
Present	203	53 (26·1)	1·1 (0·8-1·4)	0·299	-	-
Others	3617	832 (23·0)	1	-	-	-
Radiology database	-	-	-	-	-	-
Present	92	13 (13·7)	0.5 (0·3-0·9)	0·038*	0.7 (0·4-1·2)	0·285
Others	3725	872 (23·4)	1	-	1	-
Pharmacy database	-	-	-	-	-	-
Present	1754	341 (19·4)	0·7 (0·6-0·8)	0·000*	0·9 (0·8-1·0)	0·839
Others	2066	544 (26·3)	1	-	1	-

**Significant p value*
PR-Prevalence ratio; CI-Confidence Interval; EPTB-Extra Pulmonary Tuberculosis;

Qualitative component

A total of 22 healthcare providers from various clinical departments at the hospital were interviewed. There were ten physicians of whom seven were female. Six physicians had a work experience of > 10 years. In addition there were 12 paramedical staff including nurses, laboratory technicians and RNTCP staff most of whom had >10 years of work experience.

For peer review only - http://bmjopen.bmj.com/site/about/guidelines.xhtml

The four themes that emerged through the qualitative analysis were (i) basic diagnostic modalities and treatment promote notification of TB (ii) misconceptions regarding notification and its process are common amongst healthcare providers (iii) despite training efforts, introduction of the Nikshay portal does not ensure notification of all patients, and (iv) establishing systems for notification will go a long way in improving notifications. **(Table 3, and 4)**

Table 3. Barriers and solutions identified for TB notification at a private tertiary care teaching hospital in Bengaluru, India 2015-2016

Barriers	***Solutions***
TB patients diagnosed by culture, histopathology, radiology, BAL usually missed	Integration of LIS
Incomplete notification among inpatients	Triangulation of TB data from all possible sources
MDR TB missed	Proper documentation and communication which helps in notification.
Lack of dedicated manpower	Appointment of notification officer
Non-DOT not notified	Referral of all patients started on ATT by the treating doctor to the notification officer
Knowledge issues	Awareness about notification communicated
Lack of capacity building	Refresher trainings about Nikshay
Absence of hospital notification policy and standard operating procedure	Institutional notification policy
Inadequate networking between stakeholders	Having single notification desk with dedicated telephone number
Patient confidentiality concerns	Patient counselling about the importance of notification, ensuring adequate cyber security
Duplication of data	Unique identifier (such as social security number, in India Aadhar number) to prevent duplication that help notify, track and retain patient in care

BAL-Broncoalveolar lavage; LIS-Laboratory Information System; MDR TB-Multi Drug Resistant Tuberculosis; DOT-Directly Observed Treatment short course; ATT-Anti TB Treatment

i. Basic diagnostic modalities and treatment promote notification of TB:

Patients whose diagnosis was based on sputum microscopy and those receiving treatment through the RNTCP were more likely to be notified than those requiring complex diagnostics.

For peer review only - http://bmjopen.bmj.com/site/about/guidelines.xhtml

Patients who are sputum positive for TB bacteria are more likely to be notified

Diagnosis based on simple sputum smear microscopy was more likely to lead to notifications than patients requiring complex diagnostics such as radiography, biopsies, tissue examinations, bacteriological cultures or non-traditional laboratory diagnostics such as GenXpert MTB/RIF® and irrespective of whether these were inpatients or outpatients.

It was perceived that the RNTCP guidelines for notification restrict notification to only those patients diagnosed with MDR TB at an RNTCP accredited laboratory. Hence patients with MDR TB were not notified.

> *Confirmation from [an Intermediate Reference Laboratory (IRL)] is a must for initiating the MDR regimen, without this MDR TB patients cannot be (treated with DOT) or notified*
>
> *Paramedical staff 9*

Notifications were more likely for those diagnosed with pulmonary TB

Most referrals to the RNTCP DOT center were of patients diagnosed with pulmonary TB. Most patients with extra-pulmonary TB were prescribed ATT through the hospitals pharmacy and therefore bypassed the DOT center and hence notification.

> *Almost 85% extra-pulmonary patients don't take DOT or don't go to TBHV (who in turn notifies)*
>
> *Paramedical staff 1*

Receiving treatment through the RNTCP is synonymous with notification

Not all patients are initiated on DOT through the RNTCP. Some are prescribed ATT through the hospitals pharmacy at their own expense. As the responsibility for notification lies with

For peer review only - http://bmjopen.bmj.com/site/about/guidelines.xhtml

the DOT centre, patients not referred to the DOT centre are not notified. Few medical and paramedical personnel knew the procedure for notification of TB at the hospital.

> *The physician that reviews the patients (reports do not advise DOT) so the RNTCP staff is not aware (of the patient diagnosed with TB). At least if the patients visit the DOT center,(the RNTCP staff) will know…but 50% of the patients treated by doctor are not referred to the DOT center*
>
> *Paramedical staff 1*

> *Whoever goes to the DOT center (gets) registered and notified*
>
> *Physician 1*

ii. Misconceptions regarding notification and its process are common amongst healthcare providers:

The level of knowledge and awareness regarding notification and its systems was poor. Healthcare providers did not perceive notification as their responsibility.

Those who do not know, do not notify: Awareness could improve notification

Some healthcare providers were unaware that TB was a notifiable disease, others were unsure of the existing system for notifying TB and yet others presumed that notification was common knowledge.

> *I don't think TB is a notifiable disease, is it a notifiable disease? That means every TB patient we come across (should be) notified? And to whom should we notify?*
>
> *Physician 10*

Notification is someone else's responsibility

For peer review only - http://bmjopen.bmj.com/site/about/guidelines.xhtml

There was confusion regarding the responsibility for notification. Many healthcare providers considered notification the responsibility of the RNTCP and not of the institution. The laboratories considered notification the responsibility of the treating physician and vice versa.

> *What we assume is that, the patient will go back to the doctor, maybe the doctor has to notify it."*
>
> *Paramedical staff 5*

> *I think from the labs they notify directly, we haven't taken it on us to notify as yet*
>
> *Physician 2*

iii. Despite efforts, Nikshay does not ensure notification of all patients:

Inadequate training for using the notification portal, Nikshay and mandatory information requirements within the portal were barriers to notification.

Inadequate user training interferes with notifications via Nikshay portal

There were mixed opinions regarding notification via Nikshay portal. While some considered Nikshay easy to use, few remembered having any training to use Nikshay for notification. Regular updates within the Nikshay portal without training to handle updates also interfered with notifications.

> *Nikshay is (quite)easy, what we had seen during the Nikshay demo, seemed okay*
>
> *Physician 2*

> *There are changes that are made to the Nikshay portal… they haven't trained us adequately for it*
>
> *Paramedical staff 10*

For peer review only - http://bmjopen.bmj.com/site/about/guidelines.xhtml

Fear of compromising privacy interferes with notification

Fear of stigma from a breach in confidentiality prevents patients from sharing personal identifiers such as phone numbers. This limits entries into the notification portal due to missing information in "mandatory fields".

iv. Establishing systems for notification will go a long way in improving notifications: Notification policy, standard operating procedures, and dedicated personnel supported with innovative technologies such as hotlines and mobile applications were suggested.

Comprehensive institutional notification policy for TB- a necessity

Developing and implementing a comprehensive institutional notification policy to improve notification was suggested. This policy was expected to provide guidance for delegating responsibilities and linking the various components of the hospital information system to enable identification and notification of patients with TB.

Dedicated human resources could bridge gaps in the existing notification system

Supplementing the existing human resource for notification i.e., TBHV and LT, with a dedicated notification officer (institutional) and an RNTCP medical officer at the DOT center (via the program) who could liaison with each other was considered essential.

> *Let's say, we appoint a person with an intercom or maybe a mobile (phone) so that the physician just calls that person and (informs)…then s/he could probably follow-up the patient to (obtain) the details….*
>
> *Physician 9*

For peer review only - http://bmjopen.bmj.com/site/about/guidelines.xhtml

Linking records through a unique identification number is useful

Documenting the Hospital number in the RNTCP register and the government-issued Unique Identification Number (Aadhaar number)[15] in TB notification portal Nikshay could enable linkage while preventing duplication.

Developing innovative Information, communication, and Technology (ICT) support systems to aid notification

A *'one window'* concept, i.e., establishing a dedicated notification hotline, or a mobile phone application to feed the details of patients with TB diagnosed and treated at the hospital was suggested. Developing algorithms to shortlist those diagnosed with TB from the LIS, along with electronic linking of outpatient, inpatient, laboratory, diagnostic, and pharmacy records was considered to support universal notification.

> *We have to go electronic and we have to then integrate everything…ordering (drug prescription) online…the moment we have electronic medical records.... we would get much better way of tracking them.*
>
> *Physician 7*

For peer review only - http://bmjopen.bmj.com/site/about/guidelines.xhtml

Table 4: A brief description of the framework used in the qualitative data analysis for understanding TB notification at a private tertiary care teaching hospital in Bengaluru, India 2015-16

Thematic framework components and quotes	***Codes***	***Summary***	***Categories***	***Subthemes***	***Themes****
Technical issues associated with TB diagnosis: The genexpert lab here hasn't received accreditation. So we can't take that, even if it's positive. Since no accreditation, no treatment can be given from government.	Perception that confirmation from ILR is must for putting on MDR regimen, without which such patients cannot to notified and hence will be put on non-DOT	Inability of RNTCP staff to notify patients positive for MDR by GenXpertMTB/RIF® due to unclear instructions related to RNTCP accreditation of laboratory.	MDR TB not notified due to unclear instructions	Quality issues interfere with multi drug resistant TB notification	1
TB notification standard operating procedures: Because none of us know when to notify and how to notify, I may not have notified	Standard operating procedures involved in notification unknown	Lack of knowledge about the process of notification and assuming somebody else has to notify.	Lack of knowledge regarding notification	Notification is someone else's responsibility	2
Gaps in RNTCP notification policy: There are additions in NIKSHAY, still they haven't given us proper training	Gaps in notification	Refresher training on Nikshay has not been given to the RNTCP staff involved in notification even when new forms have been updated in the software.	Lack of basic training in Nikshay	Gaps in user training for the notification portal Nikshay	3
Technological involvement: The moment we have electronic medical records, if anyone is given ATT and is done online…we would get much better way of tracking them	Triangulation of patients diagnosed or treated from all departments	Scope for integrating electronic medical records with case diagnosis which will ease notification	Technical solutions to improve notification	Record linkage through unique identification numbers	4

**Themes 1-Traditional diagnostic procedures promote notification of TB patients; 2-Misconceptions regarding notification and its process is common in healthcare providers; 3-Despite efforts NIKSHAY does not ensure notification of all patients; 4-Establishing systems for notification will go a long way in improving notifications*
IRL-Intermediate Reference Laboratory; RNTCP-Revised National Tuberculosis Control Programme; MDR-Multi Drug Resistant; DOT-Directly Observed Treatment short course; ATT-Anti-TB Treatment;

For peer review only - http://bmjopen.bmj.com/site/about/guidelines.xhtml

DISCUSSION

The Indian private healthcare sector is estimated to cater to approximately 2/3rd of the inpatients and 3/4th of the outpatients in the country.[16] The private healthcare sector also accounts for 54% of the healthcare teaching facilities in India.[16] It is therefore not surprising that the private sector treats 2/3rds i.e., 2.2 million annually diagnosed patients with TB.[6] Despite this and India's mandatory TB notification policy, only 19% (0.3 million) of the TB notifications were from the private sector.[8] While poor knowledge, attitudes and organisational systems are considered to limit TB notifications.[12,17] Few reports have explored existing gaps in notification within the private sector. To our knowledge this is the first report on the extent of TB notification and its challenges from a private tertiary care teaching hospital in India.

As in other studies, poor awareness and attitudes along with inadequate systems limited the TB notifications at the hospital to a quarter of those diagnosed. Some private practitioners are of the opinion that notification of TB is unlikely to bring about change in prescription practices and question the need for collecting personal information that does not lead to public health action.[17] Therefore, training and sensitization of healthcare personnel for notification is recommended. Such training should focus on the benefits of notification from the public health and ethical perspective.[12] It is also essential for the RNTCP to provide annual feedback to healthcare providers of the numbers notified and how this affects policy for TB care. Additionally, obtaining feedback from private practitioners regarding the notification process is expected to boost provider morale and thereby, notifications.[10]

Linking hospital records electronically could simplify notification. This does not eliminate manual data entry into the notification portal. Software solutions that feed data to the

For peer review only - http://bmjopen.bmj.com/site/about/guidelines.xhtml

notification portal automatically could simplify notification and are currently being explored for MDR diagnostic machines.[7] Further, applying ICD-10 codes for diagnoses, commonly used within TB notification systems globally,[10] could standardise diagnoses, enable data capture through software systems and simplify notification.

The guidance for TB notification in India suggests the appointment of a TB nodal officer.[7] The TBHV who currently fulfils this role in our context is probably overburdened with responsibilities in the absence of the 'DOT centre medical officer,' a functionary the RNTCP. Reports from the private sector also indicate the need for additional human resources in the light of the volume of patients that they carter to.[18] Identifying an additional 'nodal officer' for TB notification from amongst existing institutional personnel could optimise the use of existing resources for notification.

Healthcare providers suggested innovative ICTs such as mobile applications for notification. However, the short messaging service (SMS), interactive voice calls (IVR) or phone calls to notify TB enabled by the RNTCP for notification, are not as popular as expected. Further, though the Niskhay mobile application that is underway to simplify the notification process holds promise,[19] whose effectiveness remains to be explored.

Healthcare providers elsewhere in India recommend simplifying the existing notification technology to promote uptake.[19] Regular training that includes Nikshay updates, which is much desired, but currently negligible could break existing technological barriers and enhance notification.

For peer review only

For peer review only - http://bmjopen.bmj.com/site/about/guidelines.xhtml

As the DOT center at the hospital is located within the Chest Medicine department, it is not surprising that sputum positive patients are notified. Only 17% of Chest Physicians notified TB, reflecting the gap between awareness and action.[11] However, in our study, ownership of the DOT center probably made notification a responsibility of the chest physician and enhanced their engagement with the RNTCP. Locating DOT centers within clinical departments with the largest burden of patients with TB patients to improve notifications is worth exploring.

Though the Indian Academy of Paediatrics supports TB treatment through the RNTCP[20] the proportion of children with TB notified was low, reflecting the limited involvement of paediatricians in the RNTCP. The questionable bioavailability of paediatric ATT formulations and alternate day dosing schedules are known barriers to engaging paediatricians with the RNTCP.[21] The introduction of the daily regimen with 'body weight bands' that inform dosing, has the potential to improve provider engagement with the RNTCP and improve TB notifications thereof, irrespective of the patients' age.[22] Also, creating a TB registry within each clinical department could improve department-wise notifications.

Linking patient records using a unique identification number (Aadhaar number)[15] or hospital number, and extending this system to involve the Nikshay portal could minimise duplication, simplify record and help retain patients in care. Studies indicate that patients are weary of sharing personal identifiers i.e., mobile phone numbers and address, for notification.[9,11] This necessitates sensitizing the general public of the need for mandatory disease notification through mass media campaigns and patients through counselling sessions. Further, perceived stigma prevents healthcare providers involved in notification

For peer review only - http://bmjopen.bmj.com/site/about/guidelines.xhtml

uncomfortable with obtaining personal identifiers from patients.[9,11] Mobile phone numbers and the patients address are mandatory fields in the Nikshay portal, without which notification is incomplete. Therefore, reminding healthcare providers of their obligation to obtain and report personal identifiers of patients with notifiable disease, as per the Indian Medical Council's (MCI) Regulations 2002,[23] might minimise discomfort in the light of responsibility. Simultaneously, mass media, posters and brochures placed in waiting rooms regarding notification could mitigate patients' fears with sharing personal identifiers.

Though punitive action for non-notification exists in India, it is not yet implemented.[23] As in other TB high burden countries, a recent mandate suggests that non-notification could result in heavy fines and even imprisonment.[24] In the light of Government of India's politico-administrative commitment towards TB control, punitive action is an eventuality that is best avoided. Therefore, at institutional level, enabling incentives for notification (tangible or intangible) and disincentives for non-notification ('warnings/ memos', or monetary penalty) could reinforce the importance of notification. Further, the RNTCP provides a cash incentive of 250 INR to a 'private' healthcare provider for every patient with TB notified.[25] Institutional proactiveness to ensure that its healthcare providers receive this incentive could also improve notifications.

Finally, our geneXpert equipment was acquired through the Initiative for Promoting Affordable and Quality TB tests (IPAQT) project,[26,27] and is registered under the "St John's Research Institute (SJRI)" facility as a separate entity within SJMCH Nikshay portal. This means that a person diagnosed with MDR TB can be notified only when the 'SJRI' option within the SJMCH notification portal is selected. Lack of awareness regarding this process for reporting MDR TB diagnosed at the institution interfered with notification. This

For peer review only - http://bmjopen.bmj.com/site/about/guidelines.xhtml

reflects the gaps in documentation and communication that exist within institutions when facilities are acquired in 'project mode' and subsequently handed over to institutions when the project ends. Understanding these issues at project initiation, documenting project procedures and ensuring 'complete knowledge transfer' to institutional personnel when institutions absorb such projects is necessary.

Methodological Issues

The mixed methods design with the quantitative and qualitative components validated and complemented each other. It is possible that our definition of notification overestimated the numbers notified. We were also liberal with our criteria for matching databases. However, we included all patients both diagnosed and treated at the hospital even if they availed a 'one-time' consultation. This probably also inflated the denominator minimising any overestimate.

The retrospective nature of the quantitative component, prevented changes in practice could affect the results if the study were prospective. Further, the quantitative component, based on a review of records, is limited by the quality of the data in the records, for example, we could not assess the association of the treating clinical department and treatment regimens on notification.

Participants in the qualitative component were fairly representative of the healthcare providers in the institution. This along with a thick description of the study context and methodology enables the reader to judge its applicability of the results to their context. The first author's understanding of her position as a physician and the study setting helped her contextualize the results. Sharing the results with all authors with diverse backgrounds and skills improved the interpretation of the results further improving generalizability.

For peer review only - http://bmjopen.bmj.com/site/about/guidelines.xhtml

Conclusions

The low proportions of TB notifications at the hospital call for urgent action to identify strategies that can improve notification. A combined approach from within (managerial) and outside the institution (RNTCP) is necessary. Generating awareness regarding notification and developing a comprehensive notification policy along with establishing a notification portal is essential. Supplementing this with technological innovations such as mobile applications and expanding the scope of the existing hospital information system to capture outpatient data and link patient records is essential.

We also call upon tertiary level teaching hospitals both within India and globally to evaluate the TB notifications and its barriers in their setting. Such information is hoped to support the development of evidence based strategies that enhance public private engagement for TB notification and control.

FIGURE LEGENDS

Figure 1: Flow of patients seeking TB care at a private tertiary care teaching hospital in Bengaluru, India

Figure 2: Flowchart showing various data sources and proportion of TB notified to RNTCP from a private tertiary care teaching hospital in Bengaluru, India 2015-16

CONTRIBUTORS

AS, GDS and RR conceived the study. AMK and EW alsong with AS, GDS and RR designed the protocol. NA did the literature search. AS and NA did data entry and extracted the electronic data. AS undertook the interviews. AS, AMK, RR, and TMM analysed and interpreted the data. AS drafted with support from AMK, and RR. GDS and EW critically revised the successive drafts. All authors have seen and approved the final version of this manuscript for publication.

For peer review only - http://bmjopen.bmj.com/site/about/guidelines.xhtml

ACKNOWLEDGEMENT

This research was conducted through the Structured Operational Research and Training Initiative (SORT IT), a global partnership led by the Special Program for Research and Training in Tropical Diseases at the World Health Organization (WHO/TDR). The model is based on a course developed jointly by the International Union Against Tuberculosis and Lung Disease (The Union) and Medécins sans Frontières (MSF/Doctors Without Borders). The specific SORT IT program which resulted in this publication was jointly developed and implemented by: The Union South-East Asia Office, New Delhi, India; the Center for Operational Research, The Union, Paris, France; The Union, Mandalay, Myanmar; MSF Luxembourg Operational Research (LuxOR); MSF Operational Center Brussels (MSF OCB); Institute of Medicine, University of Chester, UK; and Department of Medical Research, Ministry of Health and Sports, The Republic of The Union of Myanmar.

FUNDING

The training programme and open access publications costs were funded by the Department for International Development (DFID), UK and La FondationVeuve Emile Metz-Tesch (Luxembourg). The study is supported by grants from the Swedish Research Council and the Welcome trust/DBT India Alliance. The funders had no role in study design, data collection and analysis, decision to publish, or preparation of the manuscript. Corresponding author,AS confirms that she had fullaccess to all the data in the study and had final responsibility forthe decision to submit for publication.

DECLARATION OF INTERESTS

AS, GDS and RR are employed at the tertiary level teaching facility that was evaluated in this study.

DATA SHARING STATEMENT

Data will be made available with reasonable request from the corresponding author.

REFERENCES

1 World Health Organization. Global Tuberculosis Report. 2017. http://apps.who.int/iris/bitstream/10665/259366/1/9789241565516-eng.pdf?ua=1 (accessed 11 Dec 2017).

2 Subbaraman R, Nathavitharana RR, Satyanarayana S, *et al.* The Tuberculosis Cascade

For peer review only - http://bmjopen.bmj.com/site/about/guidelines.xhtml

of Care in India's Public Sector: A Systematic Review and Meta-analysis. *PLOS Med* 2016;**13**:e1002149. doi:10.1371/journal.pmed.1002149

3 United Nations. Sustainable Development Goals: 17 Goals to Transform Our World. 2015.http://www.un.org/sustainabledevelopment/health/ (accessed 11 Dec 2017).

4 World Health Organization. The End TB Strategy. 2014.http://www.who.int/tb/End_TB_brochure.pdf (accessed 11 Dec 2017).

5 Satyanarayana S, Nair SA, Chadha SS, *et al.* From where are tuberculosis patients accessing treatment in India? Results from a cross-sectional community based survey of 30 districts. *PLoS One* 2011;**6**:e24160. doi:10.1371/journal.pone.0024160

6 Arinaminpathy N, Batra D, Khaparde S, *et al.* The number of privately treated tuberculosis cases in India: an estimation from drug sales data. *Lancet Infect Dis* 2016;**16**:1255–60. doi:10.1016/S1473-3099(16)30259-6

7 Central TB Division,Directorate General of Health Services,Ministry of Health & Family Welfare G of I. Guidance for TB Notification in India. 2012.https://tbcindia.gov.in/showfile.php?lid=3139 (accessed 11 Dec 2017).

8 Central TB Division, Directorate General of Health Services, Ministry of Health and Family Welfare, Nirman Bhavan, New Delhi I. Annaual Status Report. 2017.https://tbcindia.gov.in/WriteReadData/TB India 2017.pdf (accessed 11 Dec 2017).

9 Philip S, Isaakidis P, Sagili KD, *et al.* "They Know, They Agree, but They Don't Do"- The Paradox of Tuberculosis Case Notification by Private Practitioners in Alappuzha District, Kerala, India. *PLoS One* 2015;**10**:e0123286. doi:10.1371/journal.pone.0123286

10 Uplekar M, Atre S, Wells WA, *et al.* Mandatory tuberculosis case notification in high tuberculosis-incidence countries: policy and practice. *Eur Respir J* 2016;**48**:1571–81. doi:10.1183/13993003.00956-2016

11 Thomas BE, Velayutham B, Thiruvengadam K, *et al.* Perceptions of Private Medical Practitioners on Tuberculosis Notification: A Study from Chennai, South India. *PLoS One* 2016;**11**:e0147579. doi:10.1371/journal.pone.0147579

12 Yeole RD, Khillare K, Chadha VK, *et al.* Tuberculosis case notification by private practitioners in Pune, India: how well are we doing? *Public Heal action* 2015;**5**:173–9. doi:10.5588/pha.15.0031

13 Ministry of Health and Family Welfare G of I. About Nikshay. Revised National Tuberculosis Control Programme. https://nikshay.gov.in/AboutNikshay.htm (accessed 27 Apr 2018).

14 Ritchie J, Lewis J, McNaughton Nicholls C, *et al. Qualitative research practice : a guide for social science students and researchers*. Sage 2014. https://books.google.co.in/books?id=EQSIAwAAQBAJ&printsec=frontcover&source=gbs_ge_summary_r&cad=0#v=onepage&q&f=false (accessed 24 Aug 2017).

15 Ministry of Law and Justice G of I. The Aadhar (Targeted Delivery of Financial and other Subsidies, Benefits and Services) Act 2016. https://uidai.gov.in/images/the_aadhaar_act_2016.pdf (accessed 15 Jan 2018).

For peer review only - http://bmjopen.bmj.com/site/about/guidelines.xhtml

16 Kumar S. Institute for Studies in Industrial Development Institute for Studies in Industrial Development PRIVATE SECTOR IN HEALTHCARE DELIVERY MARKET IN INDIA: Structure, Growth and Implications. Published Online First: 2015.http://isid.org.in (accessed 27 Apr 2018).

17 Gawde N. Do we need notification of tuberculosis? A public health perspective. *Indian J Med Ethics* 2013;**10**:56–8. doi:10.20529/IJME.2013.014

18 Satpati M, Burugina Nagaraja S, Shewade HD, *et al.* TB Notification from Private Health Sector in Delhi, India: Challenges Encountered by Programme Personnel and Private Health Care Providers. *Tuberc Res Treat* 2017;**2017**:6346892. doi:10.1155/2017/6346892

19 Govt launches mobile app to make TB notification simpler. http://www.dnaindia.com/india/report-govt-launches-mobile-app-to-make-tb-notification-simpler-1970345 (accessed 28 Apr 2018).

20 Amdekar Y. Consensus Statement on Childhood Tuberculosis WORKING GROUP ON TUBERCULOSIS, INDIAN ACADEMY OF PEDIATRICS (IAP). *INDIAN Pediatr* 2010;**41**.https://www.indianpediatrics.net/jan2010/41.pdf (accessed 28 Apr 2018).

21 Ramachandran G, Kumar AKH, Bhavani PK, *et al.* Pharmacokinetics of First-Line Antituberculosis Drugs in HIV-Infected Children with Tuberculosis Treated with Intermittent Regimens in India. *Antimicrob Agents Chemother* 2015;**59**:1162–7. doi:10.1128/AAC.04338-14

22 Central TB Division G of I. Technical and Operational GUidelines for TB Control in India: Treatment of TB. https://tbcindia.gov.in/showfile.php?lid=3219 (accessed 28 Apr 2018).

23 Medical Council of India ND. INDIAN MEDICAL COUNCIL (Professional Conduct, Etiquette and Ethics) Regulations, 2002. https://www.mciindia.org/documents/rulesAndRegulations/Ethics Regulations-2002.pdf (accessed 28 Apr 2018).

24 Ministry of Health and Family Welfare M 2018. Notification. Gazette, March 2018. http://egazette.nic.in/WriteReadData/2018/183924.pdf (accessed 28 Apr 2018).

25 Central TB Division, Directorate General of Health services, Ministry of Health and Family Welfare, Nirman Bhavan ND. National Strategic Plan for Tuberculosis Elimination 2017-2025. 2017;:110–108.https://tbcindia.gov.in/WriteReadData/NSP Draft 20.02.2017 1.pdf (accessed 26 Apr 2018).

26 An Innovative Intervention in Indian Private Sector: Initiative for Promoting Affordable Quality TB Tests (IPAQT). http://iammdelhi.com/wp-content/uploads/2017/09/IPQAT-Dr-manjot-kaur.pdf (accessed 8 May 2018).

27 Pai M. Promoting affordable and quality tuberculosis testing in India. *J Lab Physicians* 2013;**5**:1–4. doi:10.4103/0974-2727.115895

For peer review only - http://bmjopen.bmj.com/site/about/guidelines.xhtml

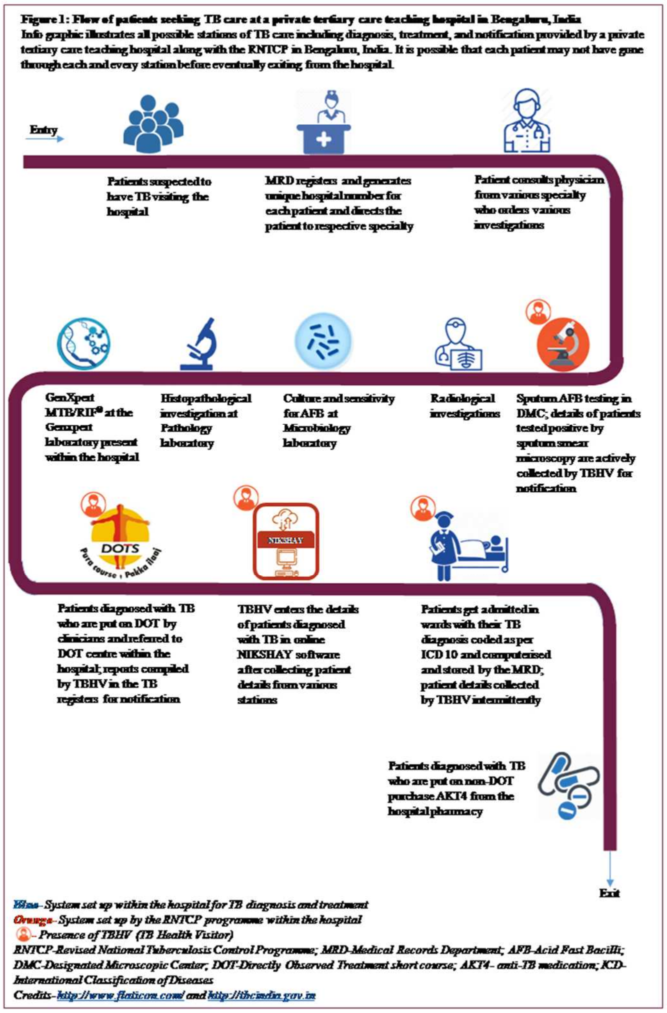

Figure 1: Flow of patients seeking TB care at a private tertiary care teaching hospital in Bengaluru, India

60x108mm (300 x 300 DPI)

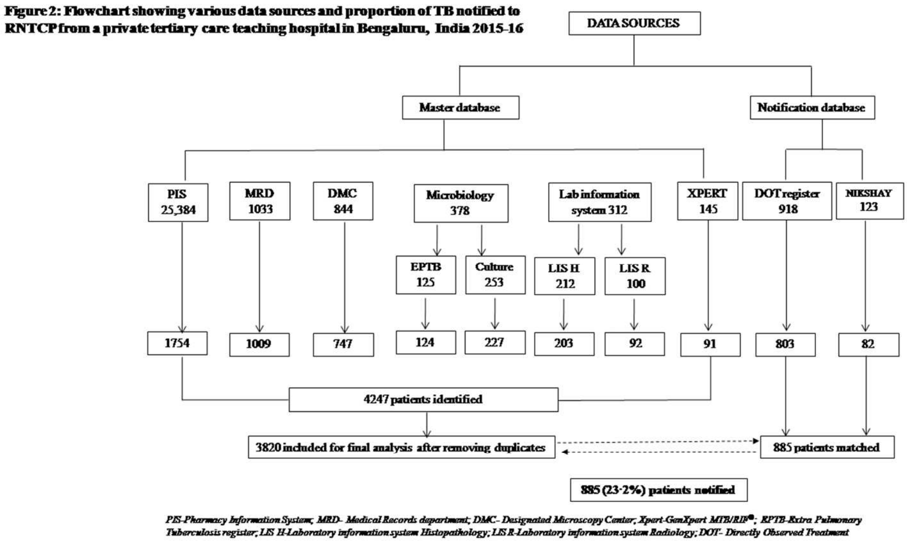

Figure 2: Flowchart showing various data sources and proportion of TB notified to RNTCP from a private tertiary care teaching hospital in Bengaluru, India 2015-16

108x60mm (300 x 300 DPI)

review only

BMJ Open

Tuberculosis Notification in a Private Tertiary Care Teaching Hospital in South India: a Mixed Methods Study

Journal:	*BMJ Open*
Manuscript ID	bmjopen-2018-023910.R1
Article Type:	Research
Date Submitted by the Author:	03-Oct-2018
Complete List of Authors:	Siddaiah, Archana; St John's National Academy of Health Sciences, Community Health<br>Ahmed, Naseer; St John's Research Institute<br>Kumar, Ajay; International Union Against Tuberculosis and Lung Disease (The Union)<br>D'Souza, George; St John's National Academy of Health Sciences<br>Wilkinson, Ewan; University of Chester<br>Maung, Thae Maung; Department of Medical Research<br>Rodrigues, Rashmi; St John's National Academy of Health Sciences, Community Health; Karolinska Institute, Public Health
**Primary Subject Heading**:	Public health
Secondary Subject Heading:	Health policy, Health services research, Infectious diseases, Public health, Qualitative research
Keywords:	operational research, TB notification, Private Medical College, Mixed Methods research, QUALITATIVE RESEARCH

SCHOLARONE™
Manuscripts

Tuberculosis Notification in a Private Tertiary Care Teaching Hospital in South India: a Mixed Methods Study

Authors

Archana Siddaiah[1], Mohammad Naseer Ahmed[2], Ajay M V Kumar[3,4], George D'Souza[5], Ewan Wilkinson[6], Thae Maung Maung[7], Rashmi Rodrigues[1,8,9]

Affiliations

[1]Department of Community Health, St John's Medical College, Bengaluru, India

[2]St John's Research Institute, Bengaluru, India

[3]International Union Against Tuberculosis and Lung Disease, Paris, France

[4]International Union Against Tuberculosis and Lung Disease, New Delhi, India

[5]Department of Pulmonary Medicine, St John's Medical College, Bengaluru, India

[6]Institute of Medicine, University of Chester, CH2 1BR, United Kingdom

[7]Department of Medical Research, Ministry of Health and Sports, Myanmar

[8]Karolinska Institute, Stockholm, Sweden

[9]Fellow, Wellcome Trust/ DBT India Alliance

Full address

Dr Archana Siddaiah, MBBS, MD
St John's Medical College Hospital
Sarjapur road, John Nagar
Kormangala
Bengaluru 560034
India
Email ID: archanapink@yahoo.com; archana.s@stjohns.in

Dr Mohammad Naseer Ahmed, MBBS
St John's Research Institute
Sarjapur road, John Nagar
Kormangala
Bengaluru
India
Email ID: ahmednaseer1992@gmail.com

Dr Ajay M V Kumar, MBBS MD
International Union Against Tuberculosis and Lung Disease (The Union)
68Boulevard Saint-Michel
F-75006 Paris
France
Email ID: akumar@theunion.org;sathyasaakshi@gmail.com
ORCID id: 0000-0003-1011-8531

For peer review only - http://bmjopen.bmj.com/site/about/guidelines.xhtml

Dr George D'Souza, MD DNB
Department of Pulmonary Medicine
St John's Medical College
SarjapurRoad, John Nagar
Kormangala
Bengaluru 560034
India
Email ID: dsouza.ga1975@gmail.com

Professor Ewan Wilkinson, MBCHB FFPH
Institute of Medicine,
Bache Hall
University of Chester
Chester
CH2 1BR
Email ID: ewanwilkinson@gmail.com
ORCID ID: 0000-0002-2167-8756

Dr Thae Maung Maung, MBBS MSc
Department of Medical Research
No.5, Ziwaka Road
Dagon Township
Yangon 11191
Myanmar
Email ID: themgmg.dr@gmail.com

Dr Rashmi Rodrigues, MBBS MD PhD
Department of Community Health
St John's Medical College
Sarjapur road, John Nagar
Kormangala
Bengaluru 560034
India
Email ID: rashmijr@gmail.com

Correspondence to:
Dr Archana Siddaiah, MBBS MD
No 321 Annex 1
St John's Medical College Hospital
Sarjapur road, John Nagar
Kormangala
Bengaluru 560034
India
Email ID: archanapink@yahoo.com; archana.s@stjohns.in
Call: (+91) 9873501269

For peer review only - http://bmjopen.bmj.com/site/about/guidelines.xhtml

Word Count:

Main text:**4649 (excluding tables)**

Summary: **299**

Number of tables: **4**

Number of figures: 2

Number of boxes: 1

Number of references: 30

Key words: operational research; notification; private medical college; mixed-methods research; qualitative research

For peer review only

For peer review only - http://bmjopen.bmj.com/site/about/guidelines.xhtml

ABSTRACT

Objectives: India contributes approximately 25% of the 'missing' cases of tuberculosis (TB) globally. Even though ~50% of patients with TB are diagnosed and treated within India's private sector, few are notified to the public healthcare system. India's TB notification policy mandates that that all TB patients are notified to Nikshay. We undertook this study in a private hospital to assess the proportion notified and the factors affecting TB notifications. We also explored the barriers and probable solutions to TB notification qualitatively from health provider's perspective.

Study setting: Private, tertiary-care, teaching hospital in Bengaluru, South India.

Methodology: This was a mixed-methods study. Quantitative component comprised a retrospective review of hospital records between 1st January 2015 and 31stDecember 2017 to determine TB notifications. Qualitative component comprised key informant interviews and focus group discussions, to elicit the barriers and facilitators of TB notification.

Results: Of 3820 patients diagnosed and treated, 885(23.2%) were notified. Notifications of sputum-smear positive patients were significantly more likely, while notifications of children with TB were less likely. Qualitative analysis yielded themes reflecting the barriers to TB notification and their solutions. Themes related to barriers were (i) basic diagnostic procedures and treatment promote notification, (ii) misconceptions regarding notification and its process is common among healthcare providers (iii) despite a national notification system, other factors have prevented notification of all patients and iv) establishing systems for notification(in the hospital) will go a long way in improving notifications.

Conclusions: Proportion of patients with TB notified by the hospital was low. A comprehensive approach both by the hospital management and the national TB programme is necessary for improving notification. This include, improving awareness among health care providers about the requirement for TB notifications, establishing a single notification portal in-hospital, digitally linking hospital records to Nikshay and designating a notification specific human resource.

Strengths and Limitations:

- A mixed methods design where the qualitative component explains and complements the findings from the quantitative component.
- Retrospective nature of the quantitative component ensured that the study procedures did not influence the notifications.
- It is likely that both the proportions notified and the number of patients diagnosed or treated is marginal overestimates.
- The findings are limited by the quality of the records maintained

BACKGROUND

For peer review only - http://bmjopen.bmj.com/site/about/guidelines.xhtml

In 2016, approximately 40% of the estimated 10.4 million tuberculosis(TB) cases were 'missing', i.e., were undiagnosed or unreported.[1]India contributes approximately25% of the 'missing' cases globally.[1,2]Finding these 'missing' cases and treating them successfully is vital to ending TB by 2030, as envisaged by the United Nations Sustainable Development Goals.[3,4]

Healthcare delivery in India involves both the public and private sectors. The Indian private healthcare sector is estimated to cater to approximately 2/3rd of the inpatients and 3/4th of the outpatients in the country.[5] The private healthcare sector also accounts for 54% of the healthcare teaching facilities in India.[5] It is therefore not surprising that approximately 2/3rd of the 2.2 million patients with TB annually are diagnosed and treated within the private healthcare sector.[6] However, in 2017 only 19% (81% from public sector) of these patients receive care from, or are notified i.e., reported, to the Revised National Tuberculosis Control Programme (RNTCP)[4,7], India's national health program for the prevention and control of TB. Though, mandatory TB notification introduced in 2012 saw a sharp increase in TB notifications, notification from the private sector continues to below.[4,7–11]. This despite launching Nikshay, the case based web based national TB notification portal, accessible to all healthcare providers, laboratories and diagnostic facilities, both public and private, nationwide.

Improving the estimates of disease prevalence though are essential for planning, monitoring, and evaluation of RNTCP. Yet barriers such as lack of time, poor awareness regarding notification, concern about breaching patient confidentiality, operational complexities in notifying along with lack of trust in the public sector. [12–14]

The information on the extent of notification from private tertiary care teaching facilities is limited. This study was designed to determine the proportion of TB cases notified and the factors that affect notification in a private tertiary care teaching healthcare facility in Karnataka State, South India. The study also explored qualitatively the gaps in the existing notification systems so as to enable the identification and development of strategies to improve notification.

METHODS

Study design

A mixed-methods study comprising retrospective review of records to quantitatively assess the proportions of patients with TB notified, and a qualitative component to identify barriers to TB notification was used.

Study setting

The study was conducted at a private tertiary level teaching hospital in Bengaluru, Karnataka state in South India. The hospital has a 1250 bedded inpatient facility and caters to approximately 2000 out patients from diverse backgrounds, everyday. A network of laboratory, pathology, and radiology services support the clinical departments at the hospital. TB specific microbiological services available are microscopy, GenXpert MTB/RIF®, solid culture, and liquid TB culture and drug susceptibility testing (DST) (such as Mycobacterial Growth Indicator Tube).

A computerised information system for these services and the pharmacy exist at the hospital. The Medical Records department (MRD) compiles and maintains inpatient and outpatient

For peer review only - http://bmjopen.bmj.com/site/about/guidelines.xhtml

hospital records in paper format. Inpatient records are available electronically and outpatient records are available in paper format.

The Indian RNTCP and its relationship with the study hospital[15]

Administrative set up: The RNTCP, a vertical national health program, strives to provide care and treatment at no cost to all patients with TB in India. The program adheres to the diagnostic and treatment recommendations of the World Health Organization (WHO)[16] The program delivers its services through a network of designated microscopy center (DMC, population covered: 0.1 million) and peripheral health institutions (PHI) (primary, secondary and tertiary healthcare facilities including all healthcare academia).

In addition, Direct observed treatment (DOT) centers at PHIs are responsible for dispensing treatment, observing treatment doses swallowed (DOT), patient follow-up and patient retention in care. Till 2017, the RNTCP followed an alternate day treatment regimen, with DOT thrice a week in the intensive phase (2 months) and weekly once in the continuation phase (4 months). All public PHIs function as DOT enters and has a TB health visitor (TBHV), responsible for DOT and patient retention. DOT centers at academic institutions however, have a medical officer in addition to the TBHV. A PHI may also function as DMC.

Private sector: The RNTCP sets guidelines but does not dictate diagnostic or treatment protocols to the private sector. However, it attempts to deliver public services to the private sector through public private partnerships (PPP) and expects all private healthcare providers to notify TB patients irrespective of a PPP, through Nikshay.

Management of TB at the hospital: By virtue of being a private tertiary care teaching hospital the RNTCP has established a DMC and a DOT center at the hospital through a PPP. The

For peer review only - http://bmjopen.bmj.com/site/about/guidelines.xhtml

RNTCP staff at the study hospital therefore, comprised an LT, an MO (position currently vacant) and a TBHV.

When diagnosed with TB at any of the clinical departments at the hospital, patients can choose to takeanti-tubercular treatment (ATT) either through the DOT center, at no cost, or through the hospital's pharmacy, for a cost. The patient's physician guides the patient's choice of treatment on a case by case basis.

Notification of patients with TB at the study hospital

Irrespective of the source of treatment, all patients with TB that are diagnosed or treated at the hospital are expected to be referred to the DOT center for registration with the RNTCP and subsequent notification via the online notification portal Nikshay. In the study hospital notification of patients with TB was the responsibility of the TBHV.

Study Population

Quantitative component

Study subjects comprised of all patients diagnosed with TB and/or treated for TB from 1stJanuary 2015 to 31stDecember 2016 comprised the study population. For this study, the definition of a patient with TB incorporated the RNTCP definitions and patients identified through pharmacy records. Pharmacy records served as a surrogate, especially for the outpatients diagnosed, in absence of outpatient electronic health records at the hospital. A patient with TB was therefore, was defined as(i) Microbiologically confirmed (RNTCP): a patient with microbiologically confirmed TB using microscopy, bacterial culture, and/or GenXpert MTB/RIF® or (ii) Clinically diagnosed (RNTCP): a patient with histopathological or radiological findings suggestive of TB, irrespective of microbiological confirmation, or

For peer review only - http://bmjopen.bmj.com/site/about/guidelines.xhtml

(iii) a patient who availed ATT from the hospital's pharmacy identified through the pharmacy information system (PIS).

Qualitative component

Health care providers caring for patients with TB from various departments including clinicians, staff nurse, researchers, LT, and TBHV were interviewed in-depth. Participants were chosen purposively to include those involved at various points within the TB case management cascade which is depicted in **Figure 1**.

Data sources, variables and data collection procedures

Quantitative component

Demographic details of patients with TB such as patient's name, date of birth, gender, education, marital status, and residence (urban/rural), and year diagnosed, clinical department visited, source of the record were extracted frommultiple sources. Data was first extracted from the inpatient electronic medical records database using the International Classification of Disease 10 (ICD 10) coding for TB (codes A15 to A19). Subsequently data from the histopathology component of the laboratory information system (LIS) was extracted. For this, search terms such as "tuberculosis", "TB" and for possible typographical errors and "lower and upper case formats" (e.g., TB or tb) were used, as these diagnoses did not follow the ICD 10 coding. Data was similarly extracted from the Radiology Information System (RIS). These comprised reports from Computerised Tomography (CT) and Magnetic Resonance Imaging (MRI). Chest radiographs were not reported in the RIS as physicians review them in the light of clinical evidence for diagnosis. A laboratory or radiology report that read "acid-fast bacilli (AFB) positive" or "MTB detected" or "strongly suggestive of TB" were considered as patients with TB. When in doubt, two physicians reviewed the reports and arrived at a

For peer review only - http://bmjopen.bmj.com/site/about/guidelines.xhtml

consensus on the diagnosis. The pharmacy information system (PIS) provided patient data for ATT purchased at the hospital's pharmacy.

Further, details of positive reports from sputum microscopy and culture registers were manually extracted and entered into Microsoft (MS) Excel as they were not available in the LIS.

A "master database" for TB patients diagnosed and/or treated in 2015and 2016 was created using the unique hospital number (allocated to a patient at registration in the hospital) to match records and delete duplicate records in the various databases (PIS, LIS, RIS and manual registers).

A "notification database" for TB patients notified was also created. For this, data from the RNTCP register at the DOT center of the hospital was entered into MS Excel. This was merged with data extracted from Nikshay portal. Patients diagnosed in late 2016 but who werenotified in the first quarter of 2017were also incorporated into this database.

In order to identify the proportions of TB cases notified the "master database" was matched with the "notification database", using the VLOOKUP function in MS Excel. The patient's name was used as the primary matching variable. Records with a typographical mismatch in the patient's name were matched using a perfect match for 'sex' within an age range of ±3 years. Flowchart of data sources is depicted in **Figure 2.**

Qualitative Component

We conducted 11in-depth interviews (IDI) with various healthcare providers and one focus group discussion (FGD) with 11 nursing staff. At the time of the study, nursing staff looked after activities such as reporting of diseases, and we conducted a FGD with them as they

For peer review only - http://bmjopen.bmj.com/site/about/guidelines.xhtml

comprised a fairly homogeneous group of female healthcare providers and were therefore included in an FGD. The first author (AS), a physician trained in qualitative research, conducted the interviews. Two of the interviews were conducted in the local language, Kannada, and the rest in English. All interviews were audio recorded. A rapporteur made field notes during the interviews. After each interview, the key points were summarized and verified with the participants for validation. Data saturation guided the sample size. Each IDI lasted for 15-45 minutes and the FGD lasted for an hour.

Data Analysis

Quantitative component

EpiData(v2.2.2.186, EpiData Association, Odense, Denmark) and Stata (v12.1, Texas, USA) software were used for data analysis. The proportion of TB patients notified was the outcome variable. Associations (unadjusted) between the outcome variable and demographic and clinical characteristics were derived using the Chi square test. All bivariate associations with a 'p value'<0.20 were included in a log-binomial regression model to obtain adjusted prevalence ratios (PR) with 95% confidence intervals. A 'p value'<0.05 was considered statistically significant.

Qualitative component

All interviews and field notes were transcribed and translated into Englishforanalysis using the 'thematic framework approach'. The first and last author (AS, RR) familiarised themselves with a few transcripts and manually coded them. The codes were then compared and categorised based on similarity. This formed the framework for the analysis.[17] The rest of the transcripts were subsequently indexed using the codes generated. Additional codes were added as and when necessary. **(Panel 1)** The data was then summarised and mapped

For peer review only - http://bmjopen.bmj.com/site/about/guidelines.xhtml

under various subthemes and themes which were reviewed by the rest of the authors for consensus.

Patient and Public Involvement Statement

Patients were not involved in the design or conduct of the study.

Ethics

Ethics approval was obtained from the Institutional Ethics Committee, St. John's Medical College, Bengaluru, Karnataka State, India and the Ethics Advisory Group of the International Union Against Tuberculosis and Lung Disease, Paris, France. Permission to conduct the study and access hospital records was obtained from the Chief of Medical Services at the hospital. Written informed consent was obtained from healthcare providers prior to interviews and included consent to audio-record the interviews.

**Panel 1: Thematic framework used for understanding the issues with TB notification at a private tertiary care teaching hospital, Bengaluru, India 2015-2016**
1.1 Gaps in the TB notification
1.2 Missing TB patients
1.3 Confidentiality issue
2. Information to doctors
3. Disease disclosure to patients
4.1 TB diagnostic standard operating procedures
4.2 Technical issues associated with TB diagnosis
5.1 Doctors' role in TB notification
5.2 Reporting of TB patients by doctors
5.3 Co-ordination between doctors and DOT center
6.1 Standard operating procedures for TB notification
6.2 Ease of notification
7.1 Policy decisions
8.1Institute's notification policy
8.2 RNTCP notification policy
8.3 Gaps in RNTCP notification policy
9.1Streamlining TB notification
9.2Technological involvement

For peer review only - http://bmjopen.bmj.com/site/about/guidelines.xhtml

DOT-Directly Observed Treatment short course; RNTCP-Revised National Tuberculosis Control Programme

RESULTS

Quantitative component

A total of 3820 patients were diagnosed with TB and/or treated during the study period. The demographic details of the patients with TB are described in **Table 1**. The median (inter-quartile range) age was 40 (27-56) years and 7% of the patients were children <15 years of age.

About a quarter of the patients received inpatient care and of them, nearly half were under the care of department of internal medicine, followed by chest medicine, neurology and paediatrics. About half of the patients with TB were identified through the pharmacy database while nearly 25% were identified through the LIS and laboratory registers.

Of the 3820 patients with TB, 885 (23.2%, 95% CI: 21.9-24.5) were notified to the RNTCP. Of those notified, only 82(9%) were also recorded in the Nikshay portal. Factors associated with notification are shown in **Table2.** Notification was significantly lower (unadjusted analysis) in children, inpatients and patients identified through the LIS and PIS. Notification was significantly higher for patients whose diagnosis was confirmed microbiologically (sputum-smear microscopy, culture or GenXpert MTB/RIF®). The final adjusted regression model showed age and sputum microscopy as determinants of notification.

For peer review only - http://bmjopen.bmj.com/site/about/guidelines.xhtml

Table 1. Demographic profile of patients with TB diagnosed and/or treated from 2015 to 2016 at a private tertiary care teaching hospital in Bengaluru, India

Variable	*Number (%)*	*Notified (%)*
Total	***3820 (100)***	***885 (23·2)***
Age in years		
0-14	264 (6·9)	24 (9·1)
15-24	476 (12·5)	118 (24·8)
25-34	802 (21·0)	166 (20·7)
35-44	670 (17·5)	159 (23·7)
45-54	598 (15·7)	160 (26·8)
55-64	503 (13·2)	129 (25·6)
65 and above	507 (13·3)	129 (25·4)
Sex		
Male	2320 (60·7)	559 (24·1)
Female	1500 (39·3)	326 (21·7)
Residence		
Within state	2362 (61·8)	567 (24·0)
Outside state	1358 (35·5)	293 (21·6)
Not available	100 (2·6)	25(25·0)
Marital status		
Unmarried	1008 (26·4)	183 (18·2)
Married	2604 (68·2)	653 (25·1)
Others	208 (5·4)	49 (23·6)
Year diagnosed		
2015	2071 (54·2)	482 (23·3)
2016	1749 (45·8)	403 (23·0)
Inpatient		
Yes	1009 (26·4)	137 (13·6)
No	2811 (73·6)	748 (26·6)
Department (n=1009)		
Medicine	484 (48·0)	64 (13·2)
Pulmonary Medicine	141 (14·0)	21 (14·9)
Paediatrics	81 (8·0)	16 (19·8)
Neurology	88 (8·7)	5 (5·7)
General Surgery	41 (4·1)	5 (12·2)
Orthopaedics	50 (5·0)	8 (16·0)
Others	124 (12·3)	15 (12·1)
Source of TB patients*		
Sputum microscopy register	747 (19·6)	481 (64·4)
Extrapulmonary TB positive register	124 (3·2)	24 (19·4)
Histopathology database	203 (5·3)	53 (26·1)
Radiology database	92 (2·5)	13 (13·7)
Pharmacy database	1754 (45·9)	341 (19·4)
Culture Register	227 (5·9)	72 (31·7)
GenXpert MTB/RIF® register	91 (2·4)	38 (41·8)
Inpatient database	1009 (26·4)	137 (13·6)

For peer review only - http://bmjopen.bmj.com/site/about/guidelines.xhtml

For peer review only

*Cumulative percentage may add up to more than 100 since one patient could have tested positive by more than one diagnostic methods

Table 2. Factors associated with TB notification at a private tertiary care teaching hospital in Bengaluru, India from 2015 to 2016

Variable	*Total*	*Notification Number (%)*	*Crude PR (95% CI)*	*P value*	*Adjusted PR (95% CI)*	*P value*
Total	3820	885 (23·2)	-	-	-	-
Age (years)	-	-	-	-	-	-
Children (<15)	264	24 (9·1)	1	-	1	-
Adults (≥ 15)	3556	861 (24·2)	2·6 (1·8-3·9)	0·000*	1·5 (1·0-2·2)	0·039*
Sex	-	-	-	-	-	-
Female	1500	326 (21·7)	1	-	-	-
Male	2320	559 (24·1)	1·1 (0·9-1·2)	-	-	-
Marital status	-	-	-	-	-	-
Unmarried	1008	183 (18·2)	1	-	1	-
Married	2604	653 (25·1)	1·3 (1·1-1·5)	0·000*	1·0 (0·9-1·2)	0·240
Others	208	49 (23·6)	1·2 (0·9-1·7)	0·066	1·1 (0·8-1·4)	0·346
Inpatient	-	-	-	-	-	-
No	2811	748 (26·6)	1	-	1	-
Yes	1009	137 (13·6)	0·4 (0·4-0·5)	0·000*	1·0 (0·8-1·2)	0·925
Residence	-	-	-	-	-	-
Within state	2362	567 (24·0)	1		-	
Outside state	1358	293 (21·6)	0·8 (0·7-1·0)	0·092	-	-
Not recorded	100	25 (25·0)	1.0 (0·7-1·4)	0·819	-	-
Year diagnosed	-	-	-	-	-	-
2015	2071	482 (23·3)	1	-	-	-
2016	1749	403 (23·0)	0·9(0·8-1·1)	0·866	-	-
Sputum smear microscopy	-	-	-	-	-	-
Positive	747	481 (64·4)	4·8 (4·4-5·4)	0·000*	4·7 (4·1-5·3)	0·000*
Others	3073	404 (13·1)	1	-	1	-
EPTB microscopy register	-	-	-	-	-	-
Positive	124	24 (19·4)	0·8 (0·5-1·1)	0·318	-	-
Others	3696	861 (23·3)	1	-	-	-
Culture	-	-	-	-	-	-
Positive	227	72 (31·7)	1·4 (1·1-1·7)	0·001*	1·0 (0·8-1·2)	0·855
Others	3593	813 (22·6)	1		1	
GenXpert MTB/RIF®	-	-	-	-	-	-
Positive	91	38 (41·8)	1·8 (1·4-2·3)	0·000*	1·1 (0·9-1·3)	0·295

For peer review only - http://bmjopen.bmj.com/site/about/guidelines.xhtml

Others	3729	847 (22·7)	1	-	1	
Histopathology database	-	-	-	-	-	-
Present	203	53 (26·1)	1·1 (0·8-1·4)	0·299	-	-
Others	3617	832 (23·0)	1	-	-	-
Radiology database	-	-	-	-	-	-
Present	92	13 (13·7)	0.5 (0·3-0·9)	0·038*	0.7 (0·4-1·2)	0·285
Others	3725	872 (23·4)	1	-	1	-
Pharmacy database	-	-	-	-	-	-
Present	1754	341 (19·4)	0·7 (0·6-0·8)	0·000*	0·9 (0·8-1·0)	0·839
Others	2066	544 (26·3)	1		1	-

**Significant p value*
PR-Prevalence ratio; CI-Confidence Interval; EPTB-Extra Pulmonary Tuberculosis;

Qualitative component

A total of 22 healthcare providers (11 from IDI and 11 from FGD) from various clinical departments at the hospital were interviewed. There were ten physicians of whom seven were female. Six physicians had a work experience of >10 years. In addition, there were 12 paramedical staff including nurses, laboratory technicians and RNTCP staff most of whom had >10 years of work experience.

The four themes that emerged through the qualitative analysis were (1) basic diagnostic modalities and treatment promote notification of TB (2) misconceptions regarding notification and its process are common amongst healthcare providers (3) despite a national notification system, other factors prevented notification of all patients, and (4) establishing systems (in the hospital) for notification will go a long way in improving notifications.**(Table 3, and 4)**

For peer review only - http://bmjopen.bmj.com/site/about/guidelines.xhtml

Table 3. Barriers and solutions identified for TB notification at a private tertiary care teaching hospital in Bengaluru, India 2015-2016

Barriers	***Solutions***
TB patients diagnosed by culture, histopathology, radiology, BAL usually missed	Integration of LIS
Incomplete notification among inpatients	Triangulation of TB data from all possible sources
MDR TB missed	Proper documentation and communication which helps in notification.
Lack of dedicated manpower	Appointment of notification officer
Non-DOT not notified	Referral of all patients started on ATT by the treating doctor to the notification officer
Knowledge issues	Awareness about notification communicated
Lack of capacity building	Refresher trainings about Nikshay
Absence of hospital notification policy and standard operating procedure	Institutional notification policy
Inadequate networking between stakeholders	Having single notification desk with dedicated telephone number
Patient confidentiality concerns	Patient counselling about the importance of notification, ensuring adequate cyber security
Duplication of data	Unique identifier (such as social security number, in India Aadhar number) to prevent duplication that help notify, track and retain patient in care

BAL-Broncho-alveolar lavage; LIS-Laboratory Information System; MDR TB-Multi Drug Resistant Tuberculosis; DOT-Directly Observed Treatment short course; ATT-Anti TB Treatment

For peer review only

1. **Basic diagnostic modalities and treatment promote notification of TB:**

   Patients whose diagnosis was based on sputum microscopy and those receiving treatment through the RNTCP were more likely to be notified than those requiring complex diagnostics.

   a. **Patients who are sputum positive for TB bacteria are more likely to be notified**

      Diagnosis based on simple sputum smear microscopy was more likely to lead to notifications than patients requiring complex diagnostics such as radiography, biopsies, tissue examinations, bacteriological cultures or non-

For peer review only - http://bmjopen.bmj.com/site/about/guidelines.xhtml

traditional laboratory diagnostics such as GenXpert MTB/RIF® and irrespective of whether these were inpatients or outpatients.

It was perceived that the RNTCP guidelines for notification restrict notification to only those patients diagnosed with MDR TB at an RNTCP accredited laboratory. Hence patients with MDR TB were not notified.

"Confirmation from [an Intermediate Reference Laboratory (IRL)] is a must for initiating the MDR regimen, without this MDR TB patients cannot be (treated with DOT) or notified"

Paramedical staff 9

b. Notifications were more likely for those diagnosed with pulmonary TB

Most referrals to the RNTCP DOT center were of patients diagnosed with pulmonary TB. Most patients with extra-pulmonary TB were prescribed ATT through the hospitals pharmacy and therefore bypassed the DOT center and hence notification.

"Almost 85% extra-pulmonary patients don't take DOT or don't go to TBHV (who in turn notifies)"

Paramedical staff 1

c. Receiving treatment through the RNTCP is synonymous with notification

Not all patients are initiated on DOT through the RNTCP.Some are prescribed ATT through the hospitals pharmacy at their own expense. As the responsibility for notification lies with the DOT centre, patients not referred to

For peer review only - http://bmjopen.bmj.com/site/about/guidelines.xhtml

the DOT centre are not notified. Few medical and paramedical personnel knew the procedure for notification of TB at the hospital.

"The physician that reviews the patients (reports do not advise DOT) so the RNTCP staff is not aware (of the patient diagnosed with TB). At least if the patients visit the DOT center,(the RNTCP staff) will know…but 50% of the patients treated by doctor are not referred to the DOT center"

Paramedical staff 1

"Whoever goes to the DOT center (gets) registered and notified"

Physician 1

2. **Misconceptions regarding notification and its process are common amongst healthcare providers:**

The level of knowledge and awareness regarding notification and its systems was poor. Healthcare providers did not perceive notification as their responsibility.

a. **Those who do not know, do not notify: Awareness could improve notification**

Some healthcare providers were unaware that TB was a notifiable disease, others were unsure of the existing system for notifying TB and yet others presumed that notification was common knowledge. Out of 22 health care providers, 14 were aware of the RNTCP requirement of notification.

For peer review only - http://bmjopen.bmj.com/site/about/guidelines.xhtml

"I don't think TB is a notifiable disease, is it a notifiable disease? That means every TB patient we come across (should be) notified? And to whom should we notify?"

Physician 10

b. Notification is someone else's responsibility

There was confusion regarding the responsibility for notification. Many healthcare providers considered notification the responsibility of the RNTCP and not of the institution. The laboratories considered notification the responsibility of the treating physician and vice versa.

"What we assume is that, the patient will go back to the doctor, maybe the doctor has to notify it."

Paramedical staff 5

"I think from the labs they notify directly, we haven't taken it on us to notify as yet"

Physician 2

3. Despite a national notification system, other factors prevented notification of all patients:

Inadequate training for using the notification portal, Nikshay and mandatory information requirements within the portal were barriers to notification.

For peer review only - http://bmjopen.bmj.com/site/about/guidelines.xhtml

a. Inadequate user training interferes with notifications via Nikshay portal

There were mixed opinions regarding notification via Nikshay portal. While some considered Nikshay easy to use, few remembered having any training to use Nikshay for notification. Regular updates within the Nikshay portal without training to handle updates also interfered with notifications.

"Nikshayis (quite) easy, what we had seen during the Nikshay demo, seemed okay"

Physician 2

"There are changes that are made to the Nikshay portal… they haven't trained us adequately for it"

Paramedical staff 10

b. Fear of compromising privacy interferes with notification

Fear of stigma from a breach in confidentiality prevents patients from sharing personal identifiers such as phone numbers. This limits entries into the notification portal due to missing information in "mandatory fields".

4. Establishing systems for notification (in the hospital) will go a long way in improving notifications:

Notification policy, standard operating procedures, and dedicated personnel supported with innovative technologies such as hotlines and mobile applications were suggested.

a. Comprehensive institutional notification policy for TB- a necessity

For peer review only - http://bmjopen.bmj.com/site/about/guidelines.xhtml

Developing and implementing a comprehensive institutional notification policy to improve notification was suggested. This policy was expected to provide guidance for delegating responsibilities and linking the various components of the hospital information system to enable identification and notification of patients with TB.

b. Dedicated human resources could bridge gaps in the existing notification system

Supplementing the existing human resource for notification i.e., TBHV and LT, with a dedicated notification officer (institutional) and an RNTCP medical officer at the DOT center (via the program) who could liaison with each other was considered essential.

"*Let's say, we appoint a person with an intercom or maybe a mobile (phone) so that the physician just calls that person and (informs)…then s/he could probably follow-up the patient to (obtain) the details….*"

Physician 9

For peer review only

c. Linking records through a unique identification number is useful

Documenting the Hospital number in the RNTCP register and the government-issued Unique Identification Number (Aadhaar number) [18] in TB notification portal Nikshay could enable linkage while preventing duplication.

d. Developing innovative Information, communication, and Technology (ICT) support systems to aid notification

For peer review only - http://bmjopen.bmj.com/site/about/guidelines.xhtml

A *'one window'* concept, i.e., establishing a dedicated notification hotline, or a mobile phone application to feed the details of patients with TB diagnosed and treated at the hospital was suggested. Developing algorithms to shortlist those diagnosed with TB from the LIS, along with electronic linking of outpatient, inpatient, laboratory, diagnostic, and pharmacy records was considered to support universal notification.

"We have to go electronic and we have to then integrate everything…ordering (drug prescription) online…the moment we have electronic medical records.... we would get much better way of tracking them"

Physician 7

DISCUSSION

Indian private healthcare sector contributes to only 1/5th to the TB notification in the country.[9] Few reports have explored existing gaps in notification within the private sector. To our knowledge this is the first report on the extent of TB notification and its challenges from a private tertiary care teaching hospital in India.

As in other studies, poor awareness and attitudes along with inadequate systems limited the TB notifications at the hospital to a quarter of those diagnosed.[19–21] Some private practitioners are of the opinion that notification of TB is unlikely to bring about change in prescription practices and question the need for collecting personal information that does not lead to public health action.[14] Therefore, training and sensitization of healthcare personnel for notification is recommended. Such training should focus on the benefits of notification from the public health and ethical perspective.[13] It is also essential for the RNTCP to provide annual feedback to healthcare providers of the numbers notified and how this affects

For peer review only - http://bmjopen.bmj.com/site/about/guidelines.xhtml

policy for TB care. Additionally, obtaining feedback from private practitioners regarding the notification process is expected to boost provider morale and thereby, notifications.[11]

Linking hospital records electronically could simplify notification. This does not eliminate manual data entry into the notification portal. Software solutions that feed data to the notification portal automatically could simplify notification and are currently being explored for MDR diagnostic machines.[8]Further, applying ICD-10 codes for diagnoses, commonly used within TB notification systems globally,[11]could standardise diagnoses, enable data capture through software systems and simplify notification.

The guidance for TB notification in India suggests the appointment of a TB nodal officer.[8] The TBHV who currently fulfils this role in our context is probably overburdened with responsibilities in the absence of the ‘DOT centre medical officer,’ a functionary the RNTCP. Reports from the private sector also indicate the need for additional human resources in the light of the volume of patients that they carter to.[22] Identifying an additional ‘nodal officer’ for TB notification from amongst existing institutional personnel could optimise the use of existing resources for notification.

Healthcare providers suggested innovative ICTs such as mobile applications for notification. However, the short messaging service (SMS), interactive voice calls (IVR) or phone calls to notify TB enabled by the RNTCP for notification, are not as popular as expected. Further, though the Niskhay mobile application that is underway to simplify the notification process holds promise,[23]whose effectiveness remains to be explored.

For peer review only - http://bmjopen.bmj.com/site/about/guidelines.xhtml

Table 4: A brief description of the framework used in the qualitative data analysis for understanding TB notification at a private tertiary care teaching hospital in Bengaluru, India 2015-16

Thematic framework components and quotes	***Codes***	***Summary***	***Categories***	***Subthemes***	***Themes****
Technical issues associated with TB diagnosis:<br><br>The GenXpert MTB/RIF® lab here hasn't received accreditation. So we can't take that, even if it's positive. Since no accreditation, no treatment can be given from government.	Perception that confirmation from ILR is must for putting on MDR regimen, without which such patients cannot to notified and hence will be put on non-DOT	Inability of RNTCP staff to notify patientspositive for MDR by GenXpertMTB/RIF® due to unclear instructions related to RNTCP accreditation of laboratory.	MDR TB not notified due to unclear instructions	Quality issues interfere with multi drug resistant TB notification	1
TB notification standard operating procedures:<br><br>Because none of us know when to notify and how to notify, I may not have notified	Standard operating procedures involved in notification unknown	Lack of knowledge about the process of notification and assuming somebody else has to notify.	Lack of knowledge regarding notification	Notification is someone else's responsibility	2
Gaps in RNTCP notification policy:<br><br>There are additions in NIKSHAY, still they haven't given us proper training	Gaps in notification	Refresher training on Nikshay has not been given to the RNTCP staff involved in notification even when new forms have been updated in the software.	Lack of basic training in Nikshay	Gaps in user training for the notification portal Nikshay	3
Technological involvement:<br><br>The moment we have electronic medical records, if anyone is given ATT and is done online…we would get much better way of tracking them	Triangulation of patients diagnosed or treated from all departments	Scope for integrating electronic medical records with case diagnosis which will ease notification	Technical solutions to improve notification	Record linkage through unique identification numbers	4

**Themes 1-Traditional diagnostic procedures promote notification of TB patients; 2-Misconceptions regarding notification and its process is common in healthcare providers; 3-Despit a national notification system, other factors prevented notification of all patients; 4-Establishing systems for notification (in the hospital) will go a long way in improving notifications*
IRL-Intermediate Reference Laboratory; RNTCP-Revised National Tuberculosis Control Programme; MDR-Multi Drug Resistant; DOT-Directly Observed Treatment short course; ATT-Anti-TB Treatment;

For peer review only

For peer review only - http://bmjopen.bmj.com/site/about/guidelines.xhtml

Healthcare providers elsewhere in India recommend simplifying the existing notification technology to promote uptake.[23] Regular training that includes Nikshay updates, which is much desired, but currently negligible could break existing technological barriers and enhance notification.

As the DOT center at the hospital is located within the Chest Medicine department, it is not surprising that sputum positive patients are notified. Only 17% of Chest Physicians notified TB, reflecting the gap between awareness and action.[12] However, in our study, ownership of the DOT center probably made notification a responsibility of the chest physician and enhanced their engagement with the RNTCP. Locating DOT centers within clinical departments with the largest burden of patients with TB patients to improve notifications is worth exploring.

Though the Indian Academy of Paediatrics supports TB treatment through the RNTCP[24] the proportion of children with TB notified was low, reflecting the limited involvement of paediatricians in the RNTCP. The questionable bioavailability of paediatric ATT formulations and alternate day dosing schedules are known barriers to engaging paediatricians with the RNTCP.[25] The introduction of the daily regimen with 'body weight bands' that inform dosing, has the potential to improve provider engagement with the RNTCP and improve TB notifications thereof, irrespective of the patients' age.[15]Also, creating a TB registry within each clinical department could improve department-wise notifications.

Linking patient records using a unique identification number (Aadhaar number) [18] or hospital number, and extending this system to involve the Nikshay portal could minimise duplication, simplify record and help retain patients in care. Studies indicate that patients are

For peer review only - http://bmjopen.bmj.com/site/about/guidelines.xhtml

weary of sharing personal identifiers i.e., mobile phone numbers and address, for notification.[10,12] This necessitates sensitizing the general public of the need for mandatory disease notification through mass media campaigns and patients through counselling sessions. Further, perceived stigma prevents healthcare providers involved in notification uncomfortable with obtaining personal identifiers from patients.[10,12] Mobile phone numbers and the patients address are mandatory fields in the Nikshay portal, without which notification is incomplete. Therefore, reminding healthcare providers of their obligation to obtain and report personal identifiers of patients with notifiable disease, as per the Indian Medical Council's (MCI) Regulations 2002,[26] might minimise discomfort in the light of responsibility. Simultaneously, mass media, posters and brochures placed in waiting rooms regarding notification could mitigate patients' fears with sharing personal identifiers.

Though punitive action for non-notification exists in India, it is not yet implemented.[26] As in other TB high burden countries, a recent mandate suggests that non-notification could result in heavy fines and even imprisonment.[27] In the light of Government of India's politico-administrative commitment towards TB control, punitive action is an eventuality that is best avoided. Therefore, at institutional level, enabling incentives for notification (tangible or intangible) and disincentives for non-notification ('warnings/ memos', or monetary penalty) could reinforce the importance of notification. Further, the RNTCP provides a cash incentive of 250 INR to a 'private' healthcare provider for every patient with TB notified.[28] Institutional proactiveness to ensure that its healthcare providers receive this incentive could also improve notifications.

Finally, testing for MDR TB cases in the study hospital was done using GenXpert MTB/RIF® equipment that was acquired through the Initiative for Promoting Affordable and Quality TB

For peer review only - http://bmjopen.bmj.com/site/about/guidelines.xhtml

tests (IPAQT) project. IPAQT aims to bring WHO approved TB tests at affordable prices to patients in private sector. [29,30] This was the only MDR TB diagnostic service available at the hospital. MDR TB cases detected through IPAQT are entered into Nikshay through a sub-portal within the hospital's primary Nikshay portal. It is only through this sub-portal that a person diagnosed with MDR TB at the hospital could be notified. A lack of awareness of this separate portal prevented notification of MDR TB diagnosed in the hospital. Understanding these issues at project initiation, documenting project procedures and ensuring 'complete knowledge transfer' to institutional personnel when institutions absorb such projects is necessary.

Methodological Issues

The mixed methods design with the quantitative and qualitative components validated and complemented each other. It is possible that our definition of notification overestimated the numbers notified. We were also liberal with our criteria for matching databases. However, we included all patients both diagnosed and treated at the hospital even if they availed a 'one-time' consultation. This probably also inflated the denominator minimising any overestimate.

The retrospective nature of the quantitative component meant that the study procedures did not influence changes in notification, as might have been observed if the study were prospective. Further, the quantitative component, based on a review of records, is limited by the quality of the data in the records, for example, we could not assess the association of the treating clinical department and treatment regimens on notification.

The study included health care providers who encountered patients with TB at different points in the hospital as represented in Fig 1, including hospital staff and RNTCP staff. Therefore, we believe that this sample is fairly representative of those health care providers

For peer review only - http://bmjopen.bmj.com/site/about/guidelines.xhtml

who manage patients with TB. This, along with a description of the study context and methodology enables the reader to judge its applicability of the results to their context. The first author's position as a physician in the study setting helped her contextualize the results. Sharing the results with all authors with diverse backgrounds and skills improved the interpretation of the results further improving generalizability.

Conclusions

The low proportions of TB notifications at the hospital call for urgent action to identify strategies that can improve notification. A combined approach from within (managerial) and outside the institution (RNTCP) is necessary. Generating awareness regarding notification and developing a comprehensive notification policy along with establishing a notification portal is essential. Supplementing this with technological innovations such as mobile applications and expanding the scope of the existing hospital information system to capture outpatient data and link patient records is essential.

We also call upon tertiary level teaching hospitals both within India and globally to evaluate the TB notifications and its barriers in their setting. Such information is hoped to support the development of evidence-based strategies that enhance public private engagement for TB notification and control.

FIGURE LEGENDS

Figure 1: Flow of patients seeking TB care at a private tertiary care teaching hospital in Bengaluru, India

Figure 2: Flowchart showing various data sources and proportion of TB notified to RNTCP from a private tertiary care teaching hospital in Bengaluru, India2015-16

For peer review only - http://bmjopen.bmj.com/site/about/guidelines.xhtml

CONTRIBUTORS

AS, GDS and RR conceived the study. AMK and EW along with AS, GDS and RR designed the protocol. NA did the literature search. AS and NA did data entry and extracted the electronic data. AS undertook the interviews. AS, AMK, RR and TMM analysed and interpreted the data. AS drafted with support from AMK, and RR. GDS and EW critically revised the successive drafts. All authors have seen and approved the final version of this manuscript for publication.

ACKNOWLEDGEMENT

This research was conducted through the Structured Operational Research and Training Initiative (SORT IT), a global partnership led by the Special Program for Research and Training in Tropical Diseases at the World Health Organization (WHO/TDR). The model is based on a course developed jointly by the International Union Against Tuberculosis and Lung Disease (The Union) and Medécins sans Frontières (MSF/Doctors Without Borders). The specific SORT IT program which resulted in this publication was jointly developed and implemented by: The Union South-East Asia Office, New Delhi, India; the Center for Operational Research, The Union, Paris, France; The Union, Mandalay, Myanmar; MSF Luxembourg Operational Research (LuxOR); MSF Operational Center Brussels (MSF OCB); Institute of Medicine, University of Chester, UK; and Department of Medical Research, Ministry of Health and Sports, The Republic of The Union of Myanmar.

FUNDING

The training programme and open access publications costs were funded by the Department for International Development (DFID), UK and La FondationVeuve Emile Metz-Tesch (Luxembourg). The study is supported by grants from the Swedish Research Council and the Welcome trust/DBT India Alliance. The funders had no role in study design, data collection and analysis, decision to publish, or preparation of the manuscript. Corresponding author,AS confirms that she had fullaccess to all the data in the study and had final responsibility forthe decision to submit for publication.

DECLARATION OF INTERESTS

AS, GDS and RR are employed at the tertiary level teaching facility that was evaluated in this study.

DATA SHARING STATEMENT

Data will be made available with reasonable request from the corresponding author.

For peer review only - http://bmjopen.bmj.com/site/about/guidelines.xhtml

REFERENCES

1 World Health Organization. Global Tuberculosis Report. 2017. http://apps.who.int/iris/bitstream/10665/259366/1/9789241565516-eng.pdf?ua=1 (accessed 11 Dec 2017).

2 Subbaraman R, Nathavitharana RR, Satyanarayana S, *et al.* The Tuberculosis Cascade of Care in India's Public Sector: A Systematic Review and Meta-analysis. *PLOS Med* 2016;**13**:e1002149. doi:10.1371/journal.pmed.1002149

3 United Nations. Sustainable Development Goals: 17 Goals to Transform Our World. 2015.http://www.un.org/sustainabledevelopment/health/ (accessed 11 Dec 2017).

4 World Health Organization. The End TB Strategy. 2014.http://www.who.int/tb/End_TB_brochure.pdf (accessed 11 Dec 2017).

5 Kumar S. Institute for Studies in Industrial Development Institute for Studies in Industrial Development PRIVATE SECTOR IN HEALTHCARE DELIVERY MARKET IN INDIA: Structure, Growth and Implications. Published Online First: 2015.http://isid.org.in (accessed 27 Apr 2018).

6 Satyanarayana S, Nair SA, Chadha SS, *et al.* From where are tuberculosis patients accessing treatment in India? Results from a cross-sectional community based survey of 30 districts. *PLoS One* 2011;**6**:e24160. doi:10.1371/journal.pone.0024160

7 Arinaminpathy N, Batra D, Khaparde S, *et al.* The number of privately treated tuberculosis cases in India: an estimation from drug sales data. *Lancet Infect Dis* 2016;**16**:1255–60. doi:10.1016/S1473-3099(16)30259-6

8 Central TB Division,Directorate General of Health Services,Ministry of Health & Family Welfare G of I. Guidance for TB Notification in India. 2012.https://tbcindia.gov.in/showfile.php?lid=3139 (accessed 11 Dec 2017).

9 Central TB Division, Directorate General of Health Services, Ministry of Health and Family Welfare, Nirman Bhavan, New Delhi I. TB India 2017. Revised National Tuberculosis Control Programme. Annual Status Report. 2017.https://tbcindia.gov.in/WriteReadData/TB India 2017.pdf (accessed 11 Dec 2017).

10 Philip S, Isaakidis P, Sagili KD, *et al.* "They Know, They Agree, but They Don't Do"- The Paradox of Tuberculosis Case Notification by Private Practitioners in Alappuzha District, Kerala, India. *PLoS One* 2015;**10**:e0123286. doi:10.1371/journal.pone.0123286

11 Uplekar M, Atre S, Wells WA, *et al.* Mandatory tuberculosis case notification in high tuberculosis-incidence countries: policy and practice. *Eur Respir J* 2016;**48**:1571–81. doi:10.1183/13993003.00956-2016

12 Thomas BE, Velayutham B, Thiruvengadam K, *et al.* Perceptions of Private Medical Practitioners on Tuberculosis Notification: A Study from Chennai, South India. *PLoS One* 2016;**11**:e0147579. doi:10.1371/journal.pone.0147579

13 Yeole RD, Khillare K, Chadha VK, *et al.* Tuberculosis case notification by private

For peer review only - http://bmjopen.bmj.com/site/about/guidelines.xhtml

practitioners in Pune, India: how well are we doing? *Public Heal action* 2015;**5**:173–9. doi:10.5588/pha.15.0031

14 Gawde N. Do we need notification of tuberculosis? A public health perspective. *Indian J Med Ethics* 2013;**10**:56–8. doi:10.20529/IJME.2013.014

15 Central TB Division G of I. Technical and Operational GUidelines for TB Control in India: Treatment of TB. https://tbcindia.gov.in/showfile.php?lid=3219 (accessed 28 Apr 2018).

16 World Health Organisation. *Definitions and reporting framework for tuberculosis – 2013 revision*. 2014. doi:WHO/HTM/TB/2013.2

17 Ritchie J, Lewis J, McNaughton Nicholls C, *et al. Qualitative research practice : a guide for social science students and researchers*. Sage 2014. https://books.google.co.in/books?id=EQSIAwAAQBAJ&printsec=frontcover&source=gbs_ge_summary_r&cad=0#v=onepage&q&f=false (accessed 24 Aug 2017).

18 Ministry of Law and Justice G of I. The Aadhar (Targeted Delivery of Financial and other Subsidies, Benefits and Services) Act 2016. https://uidai.gov.in/images/the_aadhaar_act_2016.pdf (accessed 15 Jan 2018).

19 Lal SS, Sahu S, Wares F, *et al.* Intensified scale-up of public-private mix: a systems approach to tuberculosis care and control in India. *Int J Tuberc Lung Dis* 2011;**15**:97–104.http://www.ncbi.nlm.nih.gov/pubmed/21276304 (accessed 31 Aug 2018).

20 Nagaraja SB, Achanta S, Kumar AM V., *et al.* Extending tuberculosis notification to the private sector in India: programmatic challenges? *Int J Tuberc Lung Dis* 2014;**18**:1353–6. doi:10.5588/ijtld.13.0836

21 Uplekar M, Juvekar S, Morankar S, *et al.* Tuberculosis patients and practitioners in private clinics in India. *Int J Tuberc Lung Dis* 1998;**2**:324–9.http://www.ncbi.nlm.nih.gov/pubmed/9559404 (accessed 31 Aug 2018).

22 Satpati M, Burugina Nagaraja S, Shewade HD, *et al.* TB Notification from Private Health Sector in Delhi, India: Challenges Encountered by Programme Personnel and Private Health Care Providers. *Tuberc Res Treat* 2017;**2017**:6346892. doi:10.1155/2017/6346892

23 Govt launches mobile app to make TB notification simpler. http://www.dnaindia.com/india/report-govt-launches-mobile-app-to-make-tb-notification-simpler-1970345 (accessed 28 Apr 2018).

24 Amdekar Y. Consensus Statement on Childhood Tuberculosis WORKING GROUP ON TUBERCULOSIS, INDIAN ACADEMY OF PEDIATRICS (IAP). *INDIAN Pediatr* 2010;**41**.https://www.indianpediatrics.net/jan2010/41.pdf (accessed 28 Apr 2018).

25 Ramachandran G, Kumar AKH, Bhavani PK, *et al.* Pharmacokinetics of First-Line Antituberculosis Drugs in HIV-Infected Children with Tuberculosis Treated with Intermittent Regimens in India. *Antimicrob Agents Chemother* 2015;**59**:1162–7. doi:10.1128/AAC.04338-14

26 Medical Council of India ND. INDIAN MEDICAL COUNCIL (Professional Conduct, Etiquette and Ethics) Regulations, 2002. https://www.mciindia.org/documents/rulesAndRegulations/Ethics Regulations-

For peer review only - http://bmjopen.bmj.com/site/about/guidelines.xhtml

2002.pdf (accessed 28 Apr 2018).

27 Ministry of Health and Family Welfare M 2018. Notification. Gazette, March 2018. http://egazette.nic.in/WriteReadData/2018/183924.pdf (accessed 28 Apr 2018).

28 Central TB Division, Directorate General of Health services, Ministry of Health and Family Welfare, Nirman Bhavan ND. National Strategic Plan for Tuberculosis Elimination 2017-2025. 2017;:110–108.https://tbcindia.gov.in/WriteReadData/NSP Draft 20.02.2017 1.pdf (accessed 26 Apr 2018).

29 An Innovative Intervention in Indian Private Sector: Initiative for Promoting Affordable Quality TB Tests (IPAQT). http://iammdelhi.com/wp-content/uploads/2017/09/IPQAT-Dr-manjot-kaur.pdf (accessed 8 May 2018).

30 Pai M. Promoting affordable and quality tuberculosis testing in India. *J Lab Physicians* 2013;**5**:1–4. doi:10.4103/0974-2727.115895

For peer review only

For peer review only - http://bmjopen.bmj.com/site/about/guidelines.xhtml

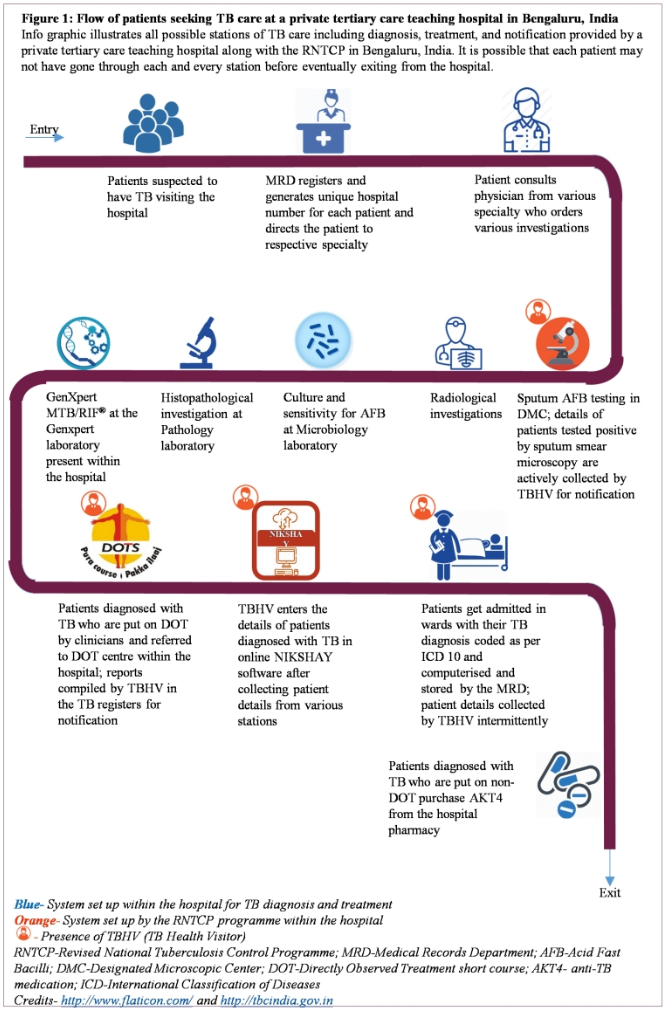

Figure 1: Flow of patients seeking TB care at a private tertiary care teaching hospital in Bengaluru, India

142x254mm (300 x 300 DPI)

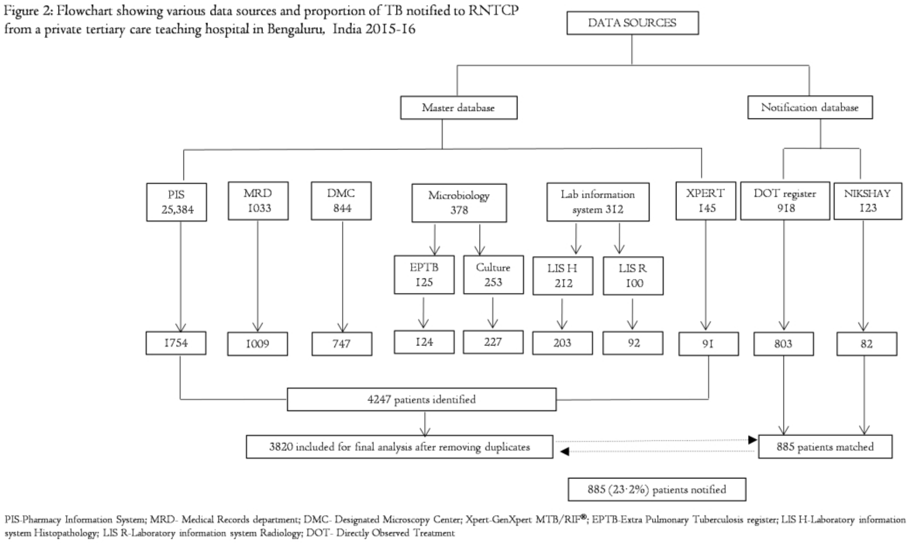

Figure 2: Flowchart showing various data sources and proportion of TB notified to RNTCP from a private tertiary care teaching hospital in Bengaluru, India2015-16

254x142mm (300 x 300 DPI)

Tuberculosis Notification in a Private Tertiary Care Teaching Hospital in South India: a Mixed Methods Study

Journal:	*BMJ Open*
Manuscript ID	bmjopen-2018-023910.R2
Article Type:	Research
Date Submitted by the Author:	31-Oct-2018
Complete List of Authors:	Siddaiah, Archana; St John's National Academy of Health Sciences, Community Health<br>Ahmed, Naseer; St John's Research Institute<br>Kumar, Ajay; International Union Against Tuberculosis and Lung Disease (The Union)<br>D'Souza, George; St John's National Academy of Health Sciences<br>Wilkinson, Ewan; University of Chester<br>Maung, Thae Maung; Department of Medical Research<br>Rodrigues, Rashmi; St John's National Academy of Health Sciences, Community Health; Karolinska Institute, Public Health
**Primary Subject Heading**:	Public health
Secondary Subject Heading:	Health policy, Health services research, Infectious diseases, Public health, Qualitative research
Keywords:	operational research, TB notification, Private Medical College, Mixed Methods research, QUALITATIVE RESEARCH

SCHOLARONE™
Manuscripts

Tuberculosis Notification in a Private Tertiary Care Teaching Hospital in South India: a Mixed Methods Study

Authors

Archana Siddaiah[1], Mohammad Naseer Ahmed[2], Ajay M V Kumar[3,4], George D'Souza[5], Ewan Wilkinson[6], Thae Maung Maung[7], Rashmi Rodrigues[1,8,9]

Affiliations

[1]Department of Community Health, St John's Medical College, Bengaluru, India

[2]St John's Research Institute, Bengaluru, India

[3]International Union Against Tuberculosis and Lung Disease, Paris, France

[4]International Union Against Tuberculosis and Lung Disease, New Delhi, India

[5]Department of Pulmonary Medicine, St John's Medical College, Bengaluru, India

[6]Institute of Medicine, University of Chester, CH2 1BR, United Kingdom

[7]Department of Medical Research, Ministry of Health and Sports, Myanmar

[8]Karolinska Institute, Stockholm, Sweden

[9]Fellow, Wellcome Trust/ DBT India Alliance

Full address

Dr Archana Siddaiah, MBBS, MD
St John's Medical College Hospital
Sarjapur road, John Nagar
Kormangala
Bengaluru 560034
India
Email ID: archanapink@yahoo.com; archana.s@stjohns.in

Dr Mohammad Naseer Ahmed, MBBS
St John's Research Institute
Sarjapur road, John Nagar
Kormangala
Bengaluru
India
Email ID: ahmednaseer1992@gmail.com

Dr Ajay M V Kumar, MBBS MD
International Union Against Tuberculosis and Lung Disease (The Union)
68Boulevard Saint-Michel
F-75006 Paris
France
Email ID: akumar@theunion.org;sathyasaakshi@gmail.com
ORCID id: 0000-0003-1011-8531

For peer review only - http://bmjopen.bmj.com/site/about/guidelines.xhtml

Dr George D'Souza, MD DNB
Department of Pulmonary Medicine
St John's Medical College
SarjapurRoad, John Nagar
Kormangala
Bengaluru 560034
India
Email ID: dsouza.ga1975@gmail.com

Professor Ewan Wilkinson, MBCHB FFPH
Institute of Medicine,
Bache Hall
University of Chester
Chester
CH2 1BR
Email ID: ewanwilkinson@gmail.com
ORCID ID: 0000-0002-2167-8756

Dr Thae Maung Maung, MBBS MSc
Department of Medical Research
No.5, Ziwaka Road
Dagon Township
Yangon 11191
Myanmar
Email ID: themgmg.dr@gmail.com

Dr Rashmi Rodrigues, MBBS MD PhD
Department of Community Health
St John's Medical College
Sarjapur road, John Nagar
Kormangala
Bengaluru 560034
India
Email ID: rashmijr@gmail.com

Correspondence to:
Dr Archana Siddaiah, MBBS MD
No 321 Annex 1
St John's Medical College Hospital
Sarjapur road, John Nagar
Kormangala
Bengaluru 560034
India
Email ID: archanapink@yahoo.com; archana.s@stjohns.in
Call: (+91) 9873501269

For peer review only - http://bmjopen.bmj.com/site/about/guidelines.xhtml

Word Count:

Main text:**4650 (excluding tables)**

Summary: **299**

Number of tables: **4**

Number of figures: 2

Number of boxes: 1

Number of references: 30

Key words: operational research; notification; private medical college; mixed-methods research; qualitative research

For peer review only

For peer review only - http://bmjopen.bmj.com/site/about/guidelines.xhtml

ABSTRACT

Objectives: India contributes approximately 25% of the 'missing' cases of tuberculosis (TB) globally. Even though ~50% of patients with TB are diagnosed and treated within India's private sector, few are notified to the public healthcare system. India's TB notification policy mandates that all TB patients are notified through Nikshay (TB notification portal) . We undertook this study in a private hospital to assess the proportion notified and factors affecting TB notifications. We explored barriers and probable solutions to TB notification qualitatively from health provider's perspective.

Study setting: Private, tertiary-care, teaching hospital in Bengaluru, South India.

Methodology: This was a mixed-methods study. Quantitative component comprised a retrospective review of hospital records between 1st January 2015 and 31stDecember 2017 to determine TB notifications. The qualitative component comprised key informant interviews and focus groups, to elicit the barriers and facilitators of TB notification.

Results: Of 3820 patients diagnosed and treated, 885(23.2%) were notified. Notifications of sputum-smear positive patients were significantly more likely, while notifications of children were less likely. Qualitative analysis yielded themes reflecting the barriers to TB notification and their solutions. Themes related to barriers were (i) basic diagnostic procedures and treatment promote notification, (ii) misconceptions regarding notification and its process is common among healthcare providers (iii) despite a national notification system, other factors have prevented notification of all patients and iv) establishing hospital systems for notification will go a long way in improving notifications.

Conclusions: The proportion of patients with TB notified by the hospital was low. A comprehensive approach both by the hospital management and the national TB programme is necessary for improving notification. This include, improving awareness among health care providers about the requirement for TB notifications, establishing a single notification portal in-hospital, digitally linking hospital records to Nikshay and designating one person to be responsible for notification.

Strengths and Limitations:

- A mixed methods design where the qualitative component explains and complements the findings from the quantitative component.
- Retrospective nature of the quantitative component ensured that the study procedures did not influence the notifications.
- It is likely that both the proportions notified and the number of patients diagnosed or treated are marginal overestimates.
- The findings are limited by the quality of the records maintained

For peer review only - http://bmjopen.bmj.com/site/about/guidelines.xhtml

BACKGROUND

In 2016, approximately 40% of the estimated 10.4 million tuberculosis(TB) cases were 'missing', i.e., were undiagnosed or unreported.[1]India contributes approximately25% of the 'missing' cases globally.[1,2]Finding these 'missing' cases and treating them successfully is vital to ending TB by 2030, as envisaged by the United Nations Sustainable Development Goals.[3,4]

Healthcare delivery in India involves both the public and private sectors. The Indian private healthcare sector is estimated to cater to approximately 2/3rd of the inpatients and 3/4th of the outpatients in the country.[5] The private healthcare sector also accounts for 54% of the healthcare teaching facilities in India.[5] It is therefore not surprising that approximately 2/3rd of the 2.2 million patients with TB annually are diagnosed and treated within the private healthcare sector.[6] However, in 2017 only 19% of these patients receive care from, or are notified i.e., reported, to the Revised National Tuberculosis Control Programme (RNTCP)[4,7], India's national health program for the prevention and control of TB, as compared to 81% from public sector. Though, mandatory TB notification introduced in 2012 saw a sharp increase in TB notifications, notification from the private sector continues to be low.[4,7–11]. This is despite launching Nikshay, the case based web based national TB notification portal, accessible to all healthcare providers, laboratories and diagnostic facilities, both public and private, nationwide.

Improving the estimates of disease prevalence though are essential for planning, monitoring, and evaluation of RNTCP. Yet barriers such as lack of time, poor awareness regarding notification, concern about breaching patient confidentiality, operational complexities in notifying along with lack of trust in the public sector prevents complete notification. [12–14]

For peer review only - http://bmjopen.bmj.com/site/about/guidelines.xhtml

The information on the extent of notification from private tertiary care teaching facilities is limited. This study was designed to determine the proportion of TB cases notified and the factors that affect notification in a private tertiary care teaching healthcare facility in Karnataka State, South India. The study also explored qualitatively the gaps in the existing notification systems so as to enable the identification and development of strategies to improve notification.

METHODS

Study design

A mixed-methods study comprising a retrospective review of records to quantitatively assess the proportions of patients with TB notified, and a qualitative component to identify barriers to TB notification was used.

Study setting

The study was conducted at a private tertiary level teaching hospital in Bengaluru, Karnataka state in South India. The hospital has 1250 beds and caters to approximately 2000 out patients daily from diverse backgrounds. A network of laboratory, pathology, and radiology services support the clinical departments at the hospital. TB specific microbiological services available are microscopy, GenXpert MTB/RIF®, solid culture, and liquid TB culture and drug susceptibility testing (DST) (such as Mycobacterial Growth Indicator Tube).

There is a computerised information system for these services and the pharmacy exist at the hospital. The Medical Records department (MRD) compiles and maintains inpatient and outpatient hospital records in paper format. Inpatient records are available electronically and outpatient records are available in paper format.

For peer review only - http://bmjopen.bmj.com/site/about/guidelines.xhtml

For peer review only

The Indian RNTCP and its relationship with the study hospital[15]

The RNTCP, a vertical national health program, strives to provide care and treatment at no cost to all patients with TB in India. The program adheres to the diagnostic and treatment recommendations of the World Health Organization (WHO)[16] The program delivers its services through a network of designated microscopy center (DMC, population covered: 0.1 million) and peripheral health institutions (PHI) (primary, secondary and tertiary healthcare facilities including all healthcare academia).

In addition, Direct observed treatment (DOT) centers at PHIs are responsible for dispensing treatment, observing treatment doses swallowed (DOT), patient follow-up and patient retention in care. Till 2017, the RNTCP followed an alternate day treatment regimen, with DOT thrice a week in the intensive phase (2 months) and weekly once in the continuation phase (4 months). All public PHIs function as DOT centers and have a TB health visitor (TBHV), responsible for DOT and patient retention. DOT centers at academic institutions however, have a medical officer in addition to the TBHV. A PHI may also function as DMC.

Even though the RNTCP sets guidelines it does not dictate diagnostic or treatment protocols to the private sector. However, it attempts to deliver public services to the private sector through public private partnerships (PPP) and expects all private healthcare providers to notify TB patients irrespective of a PPP, through Nikshay.

Management of TB at the hospital: By virtue of being a private tertiary care teaching hospital the RNTCP has established a DMC and a DOT center at the hospital through a PPP. The RNTCP staff at the study hospital therefore, comprised an LT, an MO (position currently vacant) and a TBHV.

For peer review only - http://bmjopen.bmj.com/site/about/guidelines.xhtml

When diagnosed with TB at any of the clinical departments at the hospital, patients can choose to takeanti-tubercular treatment (ATT) either through the DOT center, at no cost, or through the hospital's pharmacy, for a cost. The patient's physician guides the patient's choice of treatment on a case by case basis.

Notification of patients with TB at the study hospital

Irrespective of the source of treatment, all patients with TB that are diagnosed or treated at the hospital are expected to be referred to the DOT centers for registration with the RNTCP and subsequent notification via the online notification portal Nikshay. In the study hospital notification of patients with TB was the responsibility of the TBHV.

Study Population

Quantitative component

Study subjects comprised of all patients diagnosed with TB and/or treated for TB from 1stJanuary 2015 to 31stDecember 2016 comprised the study population. For this study, the definition of a patient with TB incorporated the RNTCP definitions and patients identified through pharmacy records. Pharmacy records served as a surrogate, especially for the outpatients diagnosed, in absence of outpatient electronic health records at the hospital. A patient with TB was therefore, defined as(i) Microbiologically confirmed (RNTCP): a patient with microbiologically confirmed TB using microscopy, bacterial culture, and/or GenXpert MTB/RIF® or (ii) Clinically diagnosed (RNTCP): a patient with histopathological or radiological findings suggestive of TB, irrespective of microbiological confirmation, or (iii) a patient who availed ATT from the hospital's pharmacy identified through the pharmacy information system (PIS).

Qualitative component

For peer review only - http://bmjopen.bmj.com/site/about/guidelines.xhtml

Health care providers caring for patients with TB from various departments including clinicians, staff nurse, researchers, LT, and TBHV were interviewed in-depth. Participants were chosen purposively to include those involved at various points within the TB case management cascade which is depicted in **Figure 1**.

Data sources, variables and data collection procedures

Quantitative component

Demographic details of patients with TB such as patient's name, date of birth, gender, education, marital status, and residence (urban/rural), and year diagnosed, clinical department visited, source of the record were extracted frommultiple sources. Data was first extracted from the inpatient electronic medical records database using the International Classification of Disease 10 (ICD 10) coding for TB (codes A15 to A19). Subsequently data from the histopathology component of the laboratory information system (LIS) was extracted. For this, search terms such as "tuberculosis", "TB" and for possible typographical errors and "lower and upper case formats" (e.g., TB or tb) were used, as these diagnoses did not follow the ICD 10 coding. Data was similarly extracted from the Radiology Information System (RIS). These comprised reports from Computerised Tomography (CT) and Magnetic Resonance Imaging (MRI). Chest radiographs were not reported in the RIS as physicians review them in the light of clinical evidence for diagnosis. A laboratory or radiology report that read "acid-fast bacilli (AFB) positive" or "MTB detected" or "strongly suggestive of TB" were considered as patients with TB. When in doubt, two physicians reviewed the reports and arrived at a consensus on the diagnosis. The pharmacy information system (PIS) provided patient data for ATT purchased at the hospital's pharmacy.

For peer review only - http://bmjopen.bmj.com/site/about/guidelines.xhtml

Further, details of positive reports from sputum microscopy and culture registers were manually extracted and entered into Microsoft (MS) Excel as they were not available in the LIS.

A "master database" for TB patients diagnosed and/or treated in 2015and 2016 was created using the unique hospital number (allocated to a patient at registration in the hospital) to match records and delete duplicate records in the various databases (PIS, LIS, RIS and manual registers).

A "notification database" for TB patients notified was also created. For this, data from the RNTCP register at the DOT center of the hospital was entered into MS Excel. This was merged with data extracted from Nikshay portal. Patients diagnosed in late 2016 but who werenotified in the first quarter of 2017were also incorporated into this database.

In order to identify the proportions of TB cases notified the "master database" was matched with the "notification database", using the VLOOKUP function in MS Excel. The patient's name was used as the primary matching variable. Records with a typographical mismatch in the patient's name were matched using a perfect match for 'sex' within an age range of ±3 years. Flowchart of data sources is depicted in **Figure 2.**

Qualitative Component

We conducted 11in-depth interviews (IDI) with various healthcare providers and one focus group discussion (FGD) with 11 nursing staff. At the time of the study, nursing staff looked after activities such as reporting of diseases, and we conducted a FGD with them as they comprised a fairly homogeneous group of female healthcare providers and were therefore included in an FGD. The first author (AS), a physician trained in qualitative research, conducted the interviews. Two of the interviews were conducted in the local language,

For peer review only - http://bmjopen.bmj.com/site/about/guidelines.xhtml

Kannada, and the rest in English. All interviews were audio recorded. A rapporteur made field notes during the interviews. After each interview, the key points were summarized and verified with the participants for validation. Data saturation guided the sample size. Each IDI lasted for 15-45 minutes and the FGD lasted for an hour.

Data Analysis

Quantitative component

EpiData(v2.2.2.186, EpiData Association, Odense, Denmark) and Stata (v12.1, Texas, USA) software were used for data analysis. The proportion of TB patients notified was the outcome variable. Associations (unadjusted) between the outcome variable and demographic and clinical characteristics were derived using the Chi square test. All bivariate associations with a 'p value'<0.20 were included in a log-binomial regression model to obtain adjusted prevalence ratios (PR) with 95% confidence intervals. A 'p value'<0.05 was considered statistically significant.

Qualitative component

All interviews and field notes were transcribed and translated into Englishforanalysis using the 'thematic framework approach'. The first and last author (AS, RR) familiarised themselves with a few transcripts and manually coded them. The codes were then compared and categorised based on similarity. This formed the framework for the analysis.[17] The rest of the transcripts were subsequently indexed using the codes generated. Additional codes were added as and when necessary. **(Box 1)** The data was then summarised and mapped under various subthemes and themes which were reviewed by the rest of the authors for consensus.

For peer review only

Patient and Public Involvement Statement

Patients were not involved in the design or conduct of the study.

Ethics

Ethics approval was obtained from the Institutional Ethics Committee, St. John's Medical College, Bengaluru, Karnataka State, India and the Ethics Advisory Group of the International Union Against Tuberculosis and Lung Disease, Paris, France. Permission to conduct the study and access hospital records was obtained from the Chief of Medical Services at the hospital. Written informed consent was obtained from healthcare providers prior to interviews and included consent to audio-record the interviews.

Box 1: Thematic framework used for understanding the issues with TB notification at a private tertiary care teaching hospital, Bengaluru, India 2015-2016

1.1 Gaps in the TB notification
1.2 Missing TB patients
1.3 Confidentiality issue
2. Information to doctors
3. Disease disclosure to patients
4.1 TB diagnostic standard operating procedures
4.2 Technical issues associated with TB diagnosis
5.1 Doctors' role in TB notification
5.2 Reporting of TB patients by doctors
5.3 Co-ordination between doctors and DOT center
6.1 Standard operating procedures for TB notification
6.2 Ease of notification
7.1 Policy decisions
8.1Institute's notification policy
8.2 RNTCP notification policy
8.3 Gaps in RNTCP notification policy
9.1Streamlining TB notification
9.2Technological involvement

For peer review only - http://bmjopen.bmj.com/site/about/guidelines.xhtml

For peer review only

DOT-Directly Observed Treatment short course; RNTCP-Revised National Tuberculosis Control Programme

RESULTS

Quantitative component

A total of 3820 patients were diagnosed with TB and/or treated during the study period. The demographic details of the patients with TB are described in **Table 1**. The median (inter-quartile range) age was 40 (27-56) years and 7% of the patients were children <15 years of age.

About a quarter of the patients received inpatient care and of them, nearly half were under the care of department of internal medicine, followed by chest medicine, neurology and paediatrics. About half of the patients with TB were identified through the pharmacy database while nearly 25% were identified through the LIS and laboratory registers.

Of the 3820 patients with TB, 885 (23.2%, 95% CI: 21.9-24.5) were notified to the RNTCP. Of those notified, only 82(9%) were also recorded in the Nikshay portal. Factors associated with notification are shown in **Table2.** Notification was significantly lower (unadjusted analysis) in children, inpatients and patients identified through the LIS and PIS. Notification was significantly higher for patients whose diagnosis was confirmed microbiologically (sputum-smear microscopy, culture or GenXpert MTB/RIF®). The final adjusted regression model showed age and sputum microscopy as determinants of notification.

For peer review only - http://bmjopen.bmj.com/site/about/guidelines.xhtml

Table 1. Demographic profile of patients with TB diagnosed and/or treated from 2015 to 2016 at a private tertiary care teaching hospital in Bengaluru, India

Variable	*Number (%)*	*Notified (%)*
Total	***3820 (100)***	***885 (23·2)***
Age in years		
0-14	264 (6·9)	24 (9·1)
15-24	476 (12·5)	118 (24·8)
25-34	802 (21·0)	166 (20·7)
35-44	670 (17·5)	159 (23·7)
45-54	598 (15·7)	160 (26·8)
55-64	503 (13·2)	129 (25·6)
65 and above	507 (13·3)	129 (25·4)
Sex		
Male	2320 (60·7)	559 (24·1)
Female	1500 (39·3)	326 (21·7)
Residence		
Within state	2362 (61·8)	567 (24·0)
Outside state	1358 (35·5)	293 (21·6)
Not available	100 (2·6)	25(25·0)
Marital status		
Unmarried	1008 (26·4)	183 (18·2)
Married	2604 (68·2)	653 (25·1)
Others	208 (5·4)	49 (23·6)
Year diagnosed		
2015	2071 (54·2)	482 (23·3)
2016	1749 (45·8)	403 (23·0)
Inpatient		
Yes	1009 (26·4)	137 (13·6)
No	2811 (73·6)	748 (26·6)
Department (n=1009)		
Medicine	484 (48·0)	64 (13·2)
Pulmonary Medicine	141 (14·0)	21 (14·9)
Paediatrics	81 (8·0)	16 (19·8)
Neurology	88 (8·7)	5 (5·7)
General Surgery	41 (4·1)	5 (12·2)
Orthopaedics	50 (5·0)	8 (16·0)
Others	124 (12·3)	15 (12·1)
Source of TB patients*		
Sputum microscopy register	747 (19·6)	481 (64·4)
Extrapulmonary TB positive register	124 (3·2)	24 (19·4)
Histopathology database	203 (5·3)	53 (26·1)
Radiology database	92 (2·5)	13 (13·7)
Pharmacy database	1754 (45·9)	341 (19·4)
Culture Register	227 (5·9)	72 (31·7)
GenXpert MTB/RIF® register	91 (2·4)	38 (41·8)
Inpatient database	1009 (26·4)	137 (13·6)

For peer review only - http://bmjopen.bmj.com/site/about/guidelines.xhtml

For peer review only

Cumulative percentage may add up to more than 100 since one patient could have tested positive by more than one diagnostic methods

Table 2. Factors associated with TB notification at a private tertiary care teaching hospital in Bengaluru, India from 2015 to 2016

Variable	*Total*	*Notification Number (%)*	*Crude PR (95% CI)*	*P value*	*Adjusted PR (95% CI)*	*P value*
Total	3820	885 (23·2)	-	-	-	-
Age (years)	-	-	-	-	-	-
Children (<15)	264	24 (9·1)	1	-	1	-
Adults (≥ 15)	3556	861 (24·2)	2·6 (1·8-3·9)	0·000*	1·5 (1·0-2·2)	0·039*
Sex	-	-	-	-	-	-
Female	1500	326 (21·7)	1	-	-	-
Male	2320	559 (24·1)	1·1 (0·9-1·2)	-	-	-
Marital status	-	-	-	-	-	-
Unmarried	1008	183 (18·2)	1	-	1	-
Married	2604	653 (25·1)	1·3 (1·1-1·5)	0·000*	1·0 (0·9-1·2)	0·240
Others	208	49 (23·6)	1·2 (0·9-1·7)	0·066	1·1 (0·8-1·4)	0·346
Inpatient	-	-	-	-	-	-
No	2811	748 (26·6)	1	-	1	-
Yes	1009	137 (13·6)	0·4 (0·4-0·5)	0·000*	1·0 (0·8-1·2)	0·925
Residence	-	-	-	-	-	-
Within state	2362	567 (24·0)	1		-	
Outside state	1358	293 (21·6)	0·8 (0·7-1·0)	0·092	-	-
Not recorded	100	25 (25·0)	1.0 (0·7-1·4)	0·819	-	-
Year diagnosed	-	-	-	-	-	-
2015	2071	482 (23·3)	1	-	-	-
2016	1749	403 (23·0)	0·9(0·8-1·1)	0·866	-	-
Sputum smear microscopy	-	-	-	-	-	-
Positive	747	481 (64·4)	4·8 (4·4-5·4)	0·000*	4·7 (4·1-5·3)	0·000*
Others	3073	404 (13·1)	1	-	1	-
EPTB microscopy register	-	-	-	-	-	-
Positive	124	24 (19·4)	0·8 (0·5-1·1)	0·318	-	-
Others	3696	861 (23·3)	1	-	-	-
Culture	-	-	-	-	-	-
Positive	227	72 (31·7)	1·4 (1·1-1·7)	0·001*	1·0 (0·8-1·2)	0·855
Others	3593	813 (22·6)	1		1	
GenXpert MTB/RIF®	-	-	-	-	-	-

For peer review only

Positive	91	38 (41·8)	1·8 (1·4-2·3)	0·000*	1·1 (0·9-1·3)	0·295
Others	3729	847 (22·7)	1	-	1	
Histopathology database	-	-	-	-	-	-
Present	203	53 (26·1)	1·1 (0·8-1·4)	0·299	-	-
Others	3617	832 (23·0)	1	-	-	-
Radiology database	-	-	-	-	-	-
Present	92	13 (13·7)	0.5 (0·3-0·9)	0·038*	0.7 (0·4-1·2)	0·285
Others	3725	872 (23·4)	1	-	1	-
Pharmacy database	-	-	-	-	-	-
Present	1754	341 (19·4)	0·7 (0·6-0·8)	0·000*	0·9 (0·8-1·0)	0·839
Others	2066	544 (26·3)	1		1	-

**Significant p value*
PR-Prevalence ratio; CI-Confidence Interval; EPTB-Extra Pulmonary Tuberculosis;

Qualitative component

A total of 22 healthcare providers (11 from IDI and 11 from FGD) from various clinical departments at the hospital were interviewed. There were ten physicians of whom seven were female. Six physicians had a work experience of >10 years. In addition, there were 12 paramedical staff including nurses, laboratory technicians and RNTCP staff most of whom had >10 years of work experience.

The four themes that emerged through the qualitative analysis were (1) basic diagnostic modalities and treatment promote notification of TB (2) misconceptions regarding notification and its process are common amongst healthcare providers (3) despite a national notification system, other factors prevented notification of all patients, and (4) establishing hospital systems for notification will go a long way in improving notifications.**(Table 3, and 4)**

For peer review only - http://bmjopen.bmj.com/site/about/guidelines.xhtml

Table 3. Barriers and solutions identified for TB notification at a private tertiary care teaching hospital in Bengaluru, India 2015-2016

Barriers	***Solutions***
TB patients diagnosed by culture, histopathology, radiology, BAL usually missed	Integration of LIS
Incomplete notification among inpatients	Triangulation of TB data from all possible sources
MDR TB missed	Proper documentation and communication which helps in notification.
Lack of dedicated manpower	Appointment of notification officer
Non-DOT not notified	Referral of all patients started on ATT by the treating doctor to the notification officer
Knowledge issues	Awareness about notification communicated
Lack of capacity building	Refresher trainings about Nikshay
Absence of hospital notification policy and standard operating procedure	Institutional notification policy
Inadequate networking between stakeholders	Having single notification desk with dedicated telephone number
Patient confidentiality concerns	Patient counselling about the importance of notification, ensuring adequate cyber security
Duplication of data	Unique identifier (such as social security number, in India Aadhar number) to prevent duplication that help notify, track and retain patient in care

BAL-Broncho-alveolar lavage; LIS-Laboratory Information System; MDR TB-Multi Drug Resistant Tuberculosis; DOT-Directly Observed Treatment short course; ATT-Anti TB Treatment

1. **Basic diagnostic modalities and treatment promote notification of TB:**

   Patients whose diagnosis was based on sputum microscopy and those receiving treatment through the RNTCP were more likely to be notified than those requiring complex diagnostics.

   a. **Patients who are sputum positive for TB bacteria are more likely to be notified**

      Diagnosis based on simple sputum smear microscopy was more likely to lead to notifications than patients requiring complex diagnostics such as radiography, biopsies, tissue examinations, bacteriological cultures or non-

For peer review only - http://bmjopen.bmj.com/site/about/guidelines.xhtml

traditional laboratory diagnostics such as GenXpert MTB/RIF® and irrespective of whether these were inpatients or outpatients.

It was perceived that the RNTCP guidelines for notification restrict notification to only those patients diagnosed with MDR TB at an RNTCP accredited laboratory. Hence patients with MDR TB were not notified.

"Confirmation from [an Intermediate Reference Laboratory (IRL)] is a must for initiating the MDR regimen, without this MDR TB patients cannot be (treated with DOT) or notified"

Paramedical staff 9(IDI)

b. Notifications are more likely for those diagnosed with pulmonary TB

Most referrals to the RNTCP DOT center were of patients diagnosed with pulmonary TB. Most patients with extra-pulmonary TB were prescribed ATT through the hospitals pharmacy and therefore bypassed the DOT center and hence notification.

"Almost 85% extra-pulmonary patients don't take DOT or don't go to TBHV (who in turn notifies)"

Paramedical staff 1(IDI)

c. Receiving treatment through the RNTCP is synonymous with notification

Not all patients are initiated on DOT through the RNTCP. Some are prescribed ATT through the hospitals pharmacy at their own expense. As the responsibility for notification lies with the DOT centre, patients not referred to

For peer review only - http://bmjopen.bmj.com/site/about/guidelines.xhtml

the DOT centre are not notified. Few medical and paramedical personnel knew the procedure for notification of TB at the hospital.

"The physician that reviews the patients (reports do not advise DOT) so the RNTCP staff is not aware (of the patient diagnosed with TB). At least if the patients visit the DOT center,(the RNTCP staff) will know…but 50% of the patients treated by doctor are not referred to the DOT center"

Paramedical staff 1(IDI)

"Whoever goes to the DOT center (gets) registered and notified"

Physician 1(IDI)

2. **Misconceptions regarding notification and its process are common amongst healthcare providers:**

The level of knowledge and awareness regarding notification and its systems was poor. Healthcare providers did not perceive notification as their responsibility.

a. **Those who do not know, do not notify: Awareness could improve notification**

Some healthcare providers were unaware that TB was a notifiable disease, others were unsure of the existing system for notifying TB and yet others presumed that notification was common knowledge. Out of 22 health care providers, 14 were aware of the RNTCP requirement of notification.

For peer review only

For peer review only - http://bmjopen.bmj.com/site/about/guidelines.xhtml

"I don't think TB is a notifiable disease, is it a notifiable disease? That means every TB patient we come across (should be) notified? And to whom should we notify?"

Physician 10(IDI)

b. Notification is someone else's responsibility

There was confusion regarding the responsibility for notification. Many healthcare providers considered notification the responsibility of the RNTCP and not of the institution. The laboratories considered notification the responsibility of the treating physician and vice versa.

"What we assume is that, the patient will go back to the doctor, maybe the doctor has to notify it."

Paramedical staff 5(FGD)

"I think from the labs they notify directly, we haven't taken it on us to notify as yet"

Physician 2(IDI)

3. Despite a national notification system, other factors prevents notification of all patients:

Inadequate training for using the notification portal, Nikshay and mandatory information requirements within the portal were barriers to notification.

For peer review only - http://bmjopen.bmj.com/site/about/guidelines.xhtml

a. Inadequate user training interferes with notifications via Nikshay portal

There were mixed opinions regarding notification via Nikshay portal. While some considered Nikshay easy to use, few remembered having any training to use Nikshay for notification. Regular updates within the Nikshay portal without training to handle updates also interfered with notifications.

"Nikshayis (quite) easy, what we had seen during the Nikshay demo, seemed okay"

Physician 2 (IDI)

"There are changes that are made to the Nikshay portal… they haven't trained us adequately for it"

Paramedical staff 10(IDI)

b. Fear of compromising privacy interferes with notification

Fear of stigma from a breach in confidentiality prevents patients from sharing personal identifiers such as phone numbers. This limits entries into the notification portal due to missing information in "mandatory fields".

4. Establishing hospital systems for notification will go a long way in improving notifications:

Notification policy, standard operating procedures, and dedicated personnel supported with innovative technologies such as hotlines and mobile applications were suggested.

a. Comprehensive institutional notification policy for TB- a necessity

For peer review only - http://bmjopen.bmj.com/site/about/guidelines.xhtml

Developing and implementing a comprehensive institutional notification policy to improve notification was suggested. This policy was expected to provide guidance for delegating responsibilities and linking the various components of the hospital information system to enable identification and notification of patients with TB.

b. Dedicated human resources could bridge gaps in the existing notification system

Supplementing the existing human resource for notification i.e., TBHV and LT, with a dedicated notification officer (institutional) and an RNTCP medical officer at the DOT center (via the program) who could liaison with each other was considered essential.

"*Let's say, we appoint a person with an intercom or maybe a mobile (phone) so that the physician just calls that person and (informs)…then s/he could probably follow-up the patient to (obtain) the details….*"

Physician 9(IDI)

c. Linking records through a unique identification number is useful

Documenting the Hospital number in the RNTCP register and the government-issued Unique Identification Number (Aadhaar number) [18] in TB notification portal Nikshay could enable linkage while preventing duplication.

d. Developing innovative Information, communication, and Technology (ICT) support systems to aid notification

For peer review only - http://bmjopen.bmj.com/site/about/guidelines.xhtml

A *'one window'* concept, i.e., establishing a dedicated notification hotline, or a mobile phone application to feed the details of patients with TB diagnosed and treated at the hospital was suggested. Developing algorithms to shortlist those diagnosed with TB from the LIS, along with electronic linking of outpatient, inpatient, laboratory, diagnostic, and pharmacy records was considered to support universal notification.

"We have to go electronic and we have to then integrate everything…ordering (drug prescription) online…the moment we have electronic medical records…. we would get much better way of tracking them"

Physician 7(IDI)

For peer review only

DISCUSSION

Indian private healthcare sector contributes to only 1/5th to the TB notification in the country.[9] Few reports have explored existing gaps in notification within the private sector. To our knowledge this is the first report on the extent of TB notification and its challenges from a private tertiary care teaching hospital in India.

As in other studies, poor awareness and attitudes along with inadequate systems limited the TB notifications at the hospital to a quarter of those diagnosed.[19–21] Some private practitioners are of the opinion that notification of TB is unlikely to bring about change in prescription practices and question the need for collecting personal information that does not lead to public health action.[14] Therefore, training and sensitization of healthcare personnel for notification is recommended. Such training should focus on the benefits of notification from the public health and ethical perspective.[13] It is also essential for the RNTCP to provide annual feedback to healthcare providers of the numbers notified and how this affects

For peer review only - http://bmjopen.bmj.com/site/about/guidelines.xhtml

policy for TB care. Additionally, obtaining feedback from private practitioners regarding the notification process is expected to boost provider morale and thereby, notifications.[11]

Linking hospital records electronically could simplify notification. This does not eliminate manual data entry into the notification portal. Software solutions that feed data to the notification portal automatically could simplify notification and are currently being explored for MDR diagnostic machines.[8]Further, applying ICD-10 codes for diagnoses, commonly used within TB notification systems globally,[11]could standardise diagnoses, enable data capture through software systems and simplify notification.

The guidance for TB notification in India suggests the appointment of a TB nodal officer.[8] The TBHV who currently fulfils this role in our context is probably overburdened with responsibilities in the absence of the 'DOT centre medical officer,' a functionary the RNTCP. Reports from the private sector also indicate the need for additional human resources in the light of the volume of patients that they carter to.[22] Identifying an additional 'nodal officer' for TB notification from amongst existing institutional personnel could optimise the use of existing resources for notification.

Healthcare providers suggested innovative ICTs such as mobile applications for notification. However, the short messaging service (SMS), interactive voice calls (IVR) or phone calls to notify TB enabled by the RNTCP for notification, are not as popular as expected. Further, though the Niskhay mobile application that is underway to simplify the notification process holds promise,[23]whose effectiveness remains to be explored.

For peer review only - http://bmjopen.bmj.com/site/about/guidelines.xhtml

Table 4: A brief description of the framework used in the qualitative data analysis for understanding TB notification at a private tertiary care teaching hospital in Bengaluru, India 2015-16

Thematic framework components and quotes	***Codes***	***Summary***	***Categories***	***Subthemes***	***Themes****
Technical issues associated with TB diagnosis:<br><br>The GenXpert MTB/RIF® lab here hasn't received accreditation. So we can't take that, even if it's positive. Since no accreditation, no treatment can be given from government.	Perception that confirmation from ILR is must for putting on MDR regimen, without which such patients cannot to notified and hence will be put on non-DOT	Inability of RNTCP staff to notify patientspositive for MDR by GenXpertMTB/RIF® due to unclear instructions related to RNTCP accreditation of laboratory.	MDR TB not notified due to unclear instructions	Quality issues interfere with multi drug resistant TB notification	1
TB notification standard operating procedures:<br><br>Because none of us know when to notify and how to notify, I may not have notified	Standard operating procedures involved in notification unknown	Lack of knowledge about the process of notification and assuming somebody else has to notify.	Lack of knowledge regarding notification	Notification is someone else's responsibility	2
Gaps in RNTCP notification policy:<br><br>There are additions in NIKSHAY, still they haven't given us proper training	Gaps in notification	Refresher training on Nikshay has not been given to the RNTCP staff involved in notification even when new forms have been updated in the software.	Lack of basic training in Nikshay	Gaps in user training for the notification portal Nikshay	3
Technological involvement:<br><br>The moment we have electronic medical records, if anyone is given ATT and is done online…we would get much better way of tracking them	Triangulation of patients diagnosed or treated from all departments	Scope for integrating electronic medical records with case diagnosis which will ease notification	Technical solutions to improve notification	Record linkage through unique identification numbers	4

**Themes 1-Traditional diagnostic procedures promote notification of TB patients; 2-Misconceptions regarding notification and its process is common in healthcare providers; 3-Despit a national notification system, other factors prevented notification of all patients; 4-Establishing hospital systems for notification will go a long way in improving notifications*
IRL-Intermediate Reference Laboratory; RNTCP-Revised National Tuberculosis Control Programme; MDR-Multi Drug Resistant; DOT-Directly Observed Treatment short course; ATT-Anti-TB Treatment;

For peer review only - http://bmjopen.bmj.com/site/about/guidelines.xhtml

Healthcare providers elsewhere in India recommend simplifying the existing notification technology to promote uptake.[23] Regular training that includes Nikshay updates, was widely requested, but currently negligible could remove existing technological barriers and enhance notification.

As the DOT center at the hospital is located within the Chest Medicine department, it is not surprising that sputum positive patients are notified. Only 17% of Chest Physicians notified TB, reflecting the gap between awareness and action.[12] However, in our study, ownership of the DOT center probably made notification a responsibility of the chest physician and enhanced their engagement with the RNTCP. Locating DOT centers within clinical departments with the largest burden of patients with TB patients to improve notifications is worth exploring.

Though the Indian Academy of Paediatrics supports TB treatment through the RNTCP[24] the proportion of children with TB notified was low, reflecting the limited involvement of paediatricians in the RNTCP. The questionable bioavailability of paediatric ATT formulations and alternate day dosing schedules are known barriers to engaging paediatricians with the RNTCP.[25] The introduction of the daily regimen with 'body weight bands' that inform dosing, has the potential to improve provider engagement with the RNTCP and improve TB notifications thereof, irrespective of the patients' age.[15]Also, creating a TB registry within each clinical department could improve department-wise notifications.

Linking patient records using a unique identification number (Aadhaar number) [18] or hospital number, and extending this system to involve the Nikshay portal could minimise duplication, simplify record and help retain patients in care. Studies indicate that patients are

For peer review only - http://bmjopen.bmj.com/site/about/guidelines.xhtml

weary of sharing personal identifiers i.e., mobile phone numbers and address, for notification.[10,12] This necessitates sensitizing the general public of the need for mandatory disease notification through mass media campaigns and patients through counselling sessions. Further, perceived stigma prevents healthcare providers involved in notification uncomfortable with obtaining personal identifiers from patients.[10,12] Mobile phone numbers and the patients address are mandatory fields in the Nikshay portal, without which notification is incomplete. Therefore, reminding healthcare providers of their obligation to obtain and report personal identifiers of patients with notifiable disease, as per the Indian Medical Council's (MCI) Regulations 2002,[26] might minimise discomfort in the light of responsibility. Simultaneously, mass media, posters and brochures placed in waiting rooms regarding notification could mitigate patients' fears with sharing personal identifiers.

Though punitive action for non-notification exists in India, it is not yet implemented.[26] As in other TB high burden countries, a recent mandate suggests that non-notification could result in heavy fines and even imprisonment.[27] In the light of Government of India's politico-administrative commitment towards TB control, punitive action is an eventuality that is best avoided. Therefore, at institutional level, enabling incentives for notification (tangible or intangible) and disincentives for non-notification ('warnings/ memos', or monetary penalty) could reinforce the importance of notification. Further, the RNTCP provides a cash incentive of 250 INR to a 'private' healthcare provider for every patient with TB notified.[28] Institutional proactiveness to ensure that its healthcare providers receive this incentive could also improve notifications.

Finally, testing for MDR TB cases in the study hospital was done using GenXpert MTB/RIF® equipment that was acquired through the Initiative for Promoting Affordable and Quality TB

For peer review only - http://bmjopen.bmj.com/site/about/guidelines.xhtml

tests (IPAQT) project. IPAQT aims to bring WHO approved TB tests at affordable prices to patients in private sector. [29,30] This was the only MDR TB diagnostic service available at the hospital. MDR TB cases detected through IPAQT are entered into Nikshay through a sub-portal within the hospital's primary Nikshay portal. It is only through this sub-portal that a person diagnosed with MDR TB at the hospital could be notified. A lack of awareness of this separate portal prevented notification of MDR TB diagnosed in the hospital. Understanding these issues at project initiation, documenting project procedures and ensuring 'complete knowledge transfer' to institutional personnel when institutions absorb such projects is necessary.

Methodological Issues

The mixed methods design with the quantitative and qualitative components validated and complemented each other. It is possible that our definition of notification overestimated the numbers notified. We were also liberal with our criteria for matching databases. However, we included all patients both diagnosed and treated at the hospital even if they availed a 'one-time' consultation. This probably also inflated the denominator minimising any overestimate.

The retrospective nature of the quantitative component meant that the study procedures did not influence changes in notification, as might have been observed if the study were prospective. Further, the quantitative component, based on a review of records, is limited by the quality of the data in the records, for example, we could not assess the association of the treating clinical department and treatment regimens on notification.

The study included health care providers who encountered patients with TB at different points in the hospital as represented in Fig 1, including hospital staff and RNTCP staff. Therefore, we believe that this sample is fairly representative of those health care providers

For peer review only - http://bmjopen.bmj.com/site/about/guidelines.xhtml

who manage patients with TB. This, along with a description of the study context and methodology enables the reader to judge its applicability of the results to their context. The first author's position as a physician in the study setting helped her contextualize the results. Sharing the results with all authors with diverse backgrounds and skills improved the interpretation of the results further improving generalizability.

Conclusions

The low proportions of TB notifications at the hospital call for urgent action to identify strategies that can improve notification. A combined approach from within (managerial) and outside the institution (RNTCP) is necessary. Generating awareness regarding notification and developing a comprehensive notification policy along with establishing a notification portal is essential. Supplementing this with technological innovations such as mobile applications and expanding the scope of the existing hospital information system to capture outpatient data and link patient records is essential.

We also call upon tertiary level teaching hospitals both within India and globally to evaluate the TB notifications and its barriers in their setting. Such information is hoped to support the development of evidence-based strategies that enhance public private engagement for TB notification and control.

FIGURE LEGENDS

Figure 1: Flow of patients seeking TB care at a private tertiary care teaching hospital in Bengaluru, India

Figure 2: Flowchart showing various data sources and proportion of TB notified to RNTCP from a private tertiary care teaching hospital in Bengaluru, India2015-16

For peer review only

For peer review only - http://bmjopen.bmj.com/site/about/guidelines.xhtml

CONTRIBUTORS

AS, GDS and RR conceived the study. AMK and EW along with AS, GDS and RR designed the protocol. NA did the literature search. AS and NA did data entry and extracted the electronic data. AS undertook the interviews. AS, AMK, RR and TMM analysed and interpreted the data. AS drafted with support from AMK, and RR. GDS and EW critically revised the successive drafts. All authors have seen and approved the final version of this manuscript for publication.

ACKNOWLEDGEMENT

This research was conducted through the Structured Operational Research and Training Initiative (SORT IT), a global partnership led by the Special Program for Research and Training in Tropical Diseases at the World Health Organization (WHO/TDR). The model is based on a course developed jointly by the International Union Against Tuberculosis and Lung Disease (The Union) and Medécins sans Frontières (MSF/Doctors Without Borders). The specific SORT IT program which resulted in this publication was jointly developed and implemented by: The Union South-East Asia Office, New Delhi, India; the Center for Operational Research, The Union, Paris, France; The Union, Mandalay, Myanmar; MSF Luxembourg Operational Research (LuxOR); MSF Operational Center Brussels (MSF OCB); Institute of Medicine, University of Chester, UK; and Department of Medical Research, Ministry of Health and Sports, The Republic of The Union of Myanmar.

FUNDING

The training programme and open access publications costs were funded by the Department for International Development (DFID), UK and La FondationVeuve Emile Metz-Tesch (Luxembourg). The study is supported by grants from the Swedish Research Council and the Welcome trust/DBT India Alliance. The funders had no role in study design, data collection and analysis, decision to publish, or preparation of the manuscript. Corresponding author,AS confirms that she had fullaccess to all the data in the study and had final responsibility forthe decision to submit for publication.

DECLARATION OF INTERESTS

AS, GDS and RR are employed at the tertiary level teaching facility that was evaluated in this study.

DATA SHARING STATEMENT

Data will be made available with reasonable request from the corresponding author.

For peer review only - http://bmjopen.bmj.com/site/about/guidelines.xhtml

REFERENCES

1 World Health Organization. Global Tuberculosis Report. 2017. http://apps.who.int/iris/bitstream/10665/259366/1/9789241565516-eng.pdf?ua=1 (accessed 11 Dec 2017).

2 Subbaraman R, Nathavitharana RR, Satyanarayana S, *et al.* The Tuberculosis Cascade of Care in India's Public Sector: A Systematic Review and Meta-analysis. *PLOS Med* 2016;**13**:e1002149. doi:10.1371/journal.pmed.1002149

3 United Nations. Sustainable Development Goals: 17 Goals to Transform Our World. 2015.http://www.un.org/sustainabledevelopment/health/ (accessed 11 Dec 2017).

4 World Health Organization. The End TB Strategy. 2014.http://www.who.int/tb/End_TB_brochure.pdf (accessed 11 Dec 2017).

5 Kumar S. Institute for Studies in Industrial Development Institute for Studies in Industrial Development PRIVATE SECTOR IN HEALTHCARE DELIVERY MARKET IN INDIA: Structure, Growth and Implications. Published Online First: 2015.http://isid.org.in (accessed 27 Apr 2018).

6 Satyanarayana S, Nair SA, Chadha SS, *et al.* From where are tuberculosis patients accessing treatment in India? Results from a cross-sectional community based survey of 30 districts. *PLoS One* 2011;**6**:e24160. doi:10.1371/journal.pone.0024160

7 Arinaminpathy N, Batra D, Khaparde S, *et al.* The number of privately treated tuberculosis cases in India: an estimation from drug sales data. *Lancet Infect Dis* 2016;**16**:1255–60. doi:10.1016/S1473-3099(16)30259-6

8 Central TB Division,Directorate General of Health Services,Ministry of Health & Family Welfare G of I. Guidance for TB Notification in India. 2012.https://tbcindia.gov.in/showfile.php?lid=3139 (accessed 11 Dec 2017).

9 Central TB Division, Directorate General of Health Services, Ministry of Health and Family Welfare, Nirman Bhavan, New Delhi I. TB India 2017. Revised National Tuberculosis Control Programme. Annual Status Report. 2017.https://tbcindia.gov.in/WriteReadData/TB India 2017.pdf (accessed 11 Dec 2017).

10 Philip S, Isaakidis P, Sagili KD, *et al.* "They Know, They Agree, but They Don't Do"- The Paradox of Tuberculosis Case Notification by Private Practitioners in Alappuzha District, Kerala, India. *PLoS One* 2015;**10**:e0123286. doi:10.1371/journal.pone.0123286

11 Uplekar M, Atre S, Wells WA, *et al.* Mandatory tuberculosis case notification in high tuberculosis-incidence countries: policy and practice. *Eur Respir J* 2016;**48**:1571–81. doi:10.1183/13993003.00956-2016

12 Thomas BE, Velayutham B, Thiruvengadam K, *et al.* Perceptions of Private Medical Practitioners on Tuberculosis Notification: A Study from Chennai, South India. *PLoS One* 2016;**11**:e0147579. doi:10.1371/journal.pone.0147579

13 Yeole RD, Khillare K, Chadha VK, *et al.* Tuberculosis case notification by private

For peer review only

For peer review only - http://bmjopen.bmj.com/site/about/guidelines.xhtml

practitioners in Pune, India: how well are we doing? *Public Heal action* 2015;**5**:173–9. doi:10.5588/pha.15.0031

14 Gawde N. Do we need notification of tuberculosis? A public health perspective. *Indian J Med Ethics* 2013;**10**:56–8. doi:10.20529/IJME.2013.014

15 Central TB Division G of I. Technical and Operational GUidelines for TB Control in India: Treatment of TB. https://tbcindia.gov.in/showfile.php?lid=3219 (accessed 28 Apr 2018).

16 World Health Organisation. *Definitions and reporting framework for tuberculosis – 2013 revision*. 2014. doi:WHO/HTM/TB/2013.2

17 Ritchie J, Lewis J, McNaughton Nicholls C, *et al. Qualitative research practice : a guide for social science students and researchers*. Sage 2014. https://books.google.co.in/books?id=EQSIAwAAQBAJ&printsec=frontcover&source=gbs_ge_summary_r&cad=0#v=onepage&q&f=false (accessed 24 Aug 2017).

18 Ministry of Law and Justice G of I. The Aadhar (Targeted Delivery of Financial and other Subsidies, Benefits and Services) Act 2016. https://uidai.gov.in/images/the_aadhaar_act_2016.pdf (accessed 15 Jan 2018).

19 Lal SS, Sahu S, Wares F, *et al.* Intensified scale-up of public-private mix: a systems approach to tuberculosis care and control in India. *Int J Tuberc Lung Dis* 2011;**15**:97–104.http://www.ncbi.nlm.nih.gov/pubmed/21276304 (accessed 31 Aug 2018).

20 Nagaraja SB, Achanta S, Kumar AM V., *et al.* Extending tuberculosis notification to the private sector in India: programmatic challenges? *Int J Tuberc Lung Dis* 2014;**18**:1353–6. doi:10.5588/ijtld.13.0836

21 Uplekar M, Juvekar S, Morankar S, *et al.* Tuberculosis patients and practitioners in private clinics in India. *Int J Tuberc Lung Dis* 1998;**2**:324–9.http://www.ncbi.nlm.nih.gov/pubmed/9559404 (accessed 31 Aug 2018).

22 Satpati M, Burugina Nagaraja S, Shewade HD, *et al.* TB Notification from Private Health Sector in Delhi, India: Challenges Encountered by Programme Personnel and Private Health Care Providers. *Tuberc Res Treat* 2017;**2017**:6346892. doi:10.1155/2017/6346892

23 Govt launches mobile app to make TB notification simpler. http://www.dnaindia.com/india/report-govt-launches-mobile-app-to-make-tb-notification-simpler-1970345 (accessed 28 Apr 2018).

24 Amdekar Y. Consensus Statement on Childhood Tuberculosis WORKING GROUP ON TUBERCULOSIS, INDIAN ACADEMY OF PEDIATRICS (IAP). *INDIAN Pediatr* 2010;**41**.https://www.indianpediatrics.net/jan2010/41.pdf (accessed 28 Apr 2018).

25 Ramachandran G, Kumar AKH, Bhavani PK, *et al.* Pharmacokinetics of First-Line Antituberculosis Drugs in HIV-Infected Children with Tuberculosis Treated with Intermittent Regimens in India. *Antimicrob Agents Chemother* 2015;**59**:1162–7. doi:10.1128/AAC.04338-14

26 Medical Council of India ND. INDIAN MEDICAL COUNCIL (Professional Conduct, Etiquette and Ethics) Regulations, 2002. https://www.mciindia.org/documents/rulesAndRegulations/Ethics Regulations-

For peer review only - http://bmjopen.bmj.com/site/about/guidelines.xhtml

2002.pdf (accessed 28 Apr 2018).

27 Ministry of Health and Family Welfare M 2018. Notification. Gazette, March 2018. http://egazette.nic.in/WriteReadData/2018/183924.pdf (accessed 28 Apr 2018).

28 Central TB Division, Directorate General of Health services, Ministry of Health and Family Welfare, Nirman Bhavan ND. National Strategic Plan for Tuberculosis Elimination 2017-2025. 2017;:110–108.https://tbcindia.gov.in/WriteReadData/NSP Draft 20.02.2017 1.pdf (accessed 26 Apr 2018).

29 An Innovative Intervention in Indian Private Sector: Initiative for Promoting Affordable Quality TB Tests (IPAQT). http://iammdelhi.com/wp-content/uploads/2017/09/IPQAT-Dr-manjot-kaur.pdf (accessed 8 May 2018).

30 Pai M. Promoting affordable and quality tuberculosis testing in India. *J Lab Physicians* 2013;**5**:1–4. doi:10.4103/0974-2727.115895

For peer review only

For peer review only - http://bmjopen.bmj.com/site/about/guidelines.xhtml

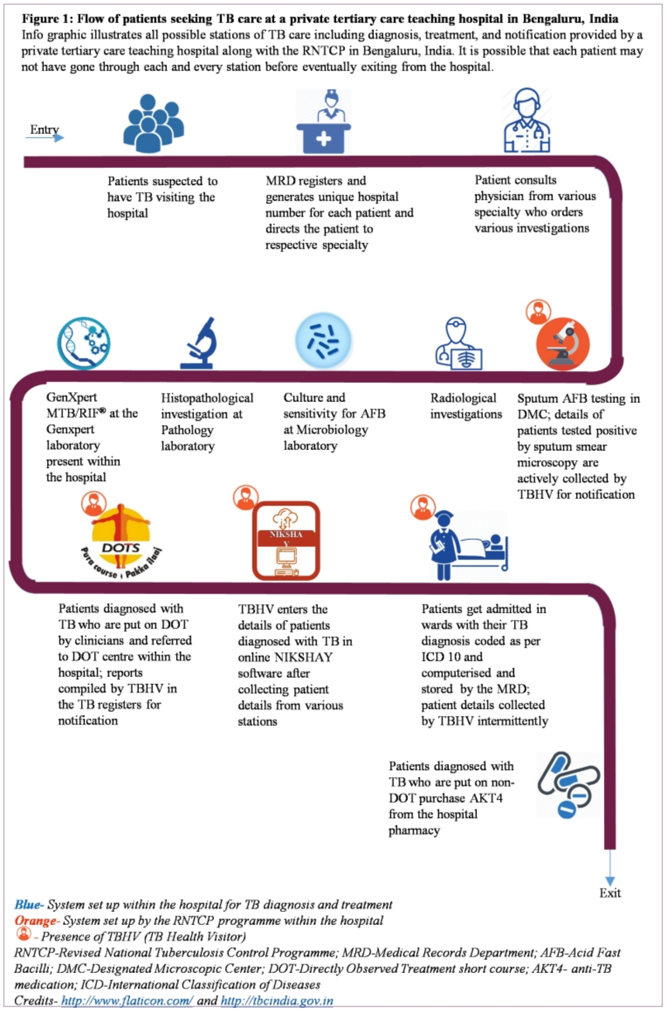

Figure 1: Flow of patients seeking TB care at a private tertiary care teaching hospital in Bengaluru, India

142x254mm (300 x 300 DPI)

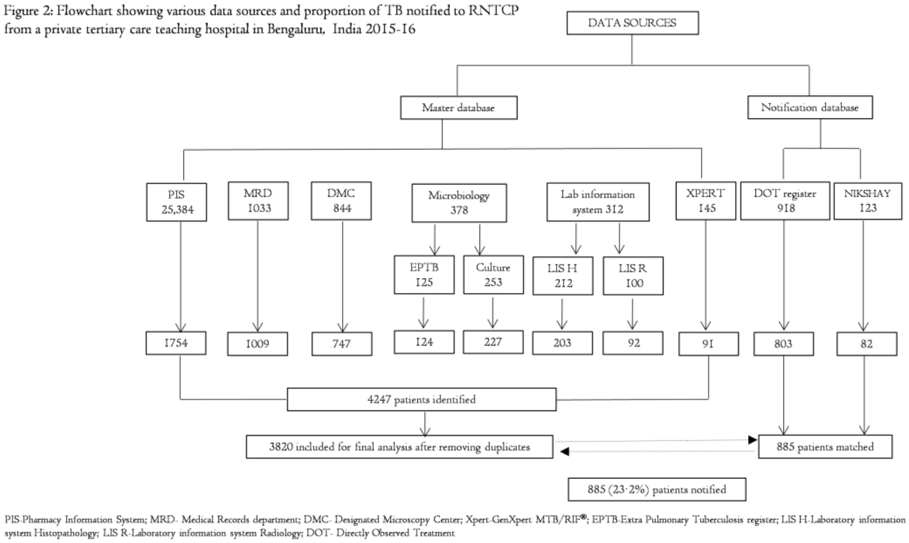

Figure 2: Flowchart showing various data sources and proportion of TB notified to RNTCP from a private tertiary care teaching hospital in Bengaluru, India2015-16

254x142mm (300 x 300 DPI)
